# Non-Coding RNAs and Oral Cancer: Small Molecules With Big Functions

**DOI:** 10.3389/fonc.2022.914593

**Published:** 2022-07-11

**Authors:** Leila Erfanparast, Mohammad Taghizadieh, Ali Akbar Shekarchi

**Affiliations:** ^1^ Department of Pediatric Dentistry, Faculty of Dentistry, Tabriz University of Medical Sciences, Tabriz, Iran; ^2^ Department of Pathology, Faculty of Medicine, Tabriz University of Medical Sciences, Tabriz, Iran; ^3^ Department of Pathology, Tabriz University of Medical Sciences, Tabriz, Iran

**Keywords:** oral cancer, non-coding RNAs, microRNAs, long non-coding RNAs, circular RNAs

## Abstract

Oral cancer remains a major public concern with considerable socioeconomic impact in the world. Despite substantial advancements have been made in treating oral cancer, the five-year survival rate for oral cancer remained undesirable, and the molecular mechanisms underlying OSCC carcinogenesis have not been fully understood. Noncoding RNAs (ncRNAs) include transfer RNAs (tRNAs), as well as small RNAs such as microRNAs, and the long ncRNAs such as HOTAIR are a large segment of the transcriptome that do not have apparent protein-coding roles, but they have been verified to play important roles in diverse biological processes, including cancer cell development. Cell death, such as apoptosis, necrosis, and autophagy, plays a vital role in the progression of cancer. A better understanding of the regulatory relationships between ncRNAs and these various types of cancer cell death is therefore urgently required. The occurrence and development of oral cancer can be controlled by increasing or decreasing the expression of ncRNAs, a method which confers broad prospects for oral cancer treatment. Therefore, it is urgent for us to understand the influence of ncRNAs on the development of different modes of oral tumor death, and to evaluate whether ncRNAs have the potential to be used as biological targets for inducing cell death and recurrence of chemotherapy. The purpose of this review is to describe the impact of ncRNAs on cell apoptosis and autophagy in oral cancer in order to explore potential targets for oral cancer therapy.

## Introduction

Oral cancer is a common and fatal malignancy among head and neck malignant neoplasms; the number of new cases of oral cancer globally was 354,864 in 2018 (1). At present, principal treatments of oral cancer include extensive exeresis of the primary carcinoma, with or without neck dissection, and pre-or postoperative adjuvant chemotherapy and radiotherapy (2). However, the overall 5-year survival rate of patients with oral cancer was 65%, and the overall 5-year survival rate of patients with advanced oral cancer was as low as 27% between 2007 and 2013 in the USA (3). Despite the application of reconstructive radical resection and postoperative radiotherapy or chemotherapy, the 5-year survival rate of patients with terminal oral cancer has not improved effectively over the past years (4–6). Furthermore, the dysphagia, maxillofacial malformation and dysarthria induced by the aforementioned therapies negatively affect the quality of life and psychology of patients (7). Therefore, there is a requirement to identify more effective treatment strategies to improve the survival rate and reduce complications of patients with oral cancer.

Programmed cell death (PCD) may balance cell death with the survival of normal cells; the equilibrium becomes disturbed and PCD plays key roles in ultimate decisions of cancer cell fate. Of note, apoptosis, autophagy, and programmed necrosis are the three main forms of PCD, easily distinguished by their morphological differences (8). Apoptosis, or type I PCD, was first described by Kerr et al. (9), and is characterized by specific morphological and biochemical changes of dying cells, including cell shrinkage, nuclear condensation and fragmentation, dynamic membrane blebbing, and loss of adhesion to neighbors or to extracellular matrix (8). Biochemical changes include chromosomal DNA cleavage into internucleosomal fragments, phosphatidylserine externalization, and a number of intracellular substrate cleavages by specific proteolysis (10, 11). Autophagy, or type II PCD, is an evolutionarily conserved catabolic process beginning with the formation of autophagosomes, double membrane-bound structures surrounding cytoplasmic macromolecules and organelles, destined for recycling (12). In general, autophagy plays a crucial pro-survival role in cell homeostasis, required during periods of starvation or stress due to growth factor deprivation (13). However, there is accumulating evidence that autophagic cells may commit suicide by undergoing cell death and coping with excessive stress, which differs from apoptosis and programmed necrosis (14). As ‘the Janus role’, autophagy controls a myriad of physiological processes including starvation, cell differentiation, cell survival and death. Besides apoptosis and autophagy, there exists a type III PCD termed programmed necrosis, which involves cell swelling, organelle dysfunction and cell lysis (15). Thus, PCD may play an important role during the preservation of tissue homoeostasis and elimination of damaged cells, – this has profound effects on malignant tissues (8).

Non-coding RNAs (ncRNAs) are transcripts that do not code for proteins, which can be roughly divided into small non-coding RNAs (smaller than 200 nt) and long non-coding RNAs (lncRNAs, longer than 200 nt) (16–25). NcRNAs account for the majority of transcriptome, interestingly the amount of ncRNAs correlates with organismal complexity (26). Emerging evidence showed that ncRNAs have regulatory roles in diverse cellular processes both in biological and pathological conditions including cancer (25, 27–33). Their roles in cancer progression are being appreciated (29, 34, 35). Among them, microRNAs (miRNAs), long non-coding RNAs (lncRNAs) and circular RNAs (circRNAs) are actively studied in recent years. Research is accelerating to decipher the underlying mechanism of ncRNA-regulated oral cancer progression (36, 37). **Table 1** shows classification of non-coding RNAs is given in.

**Table 1:** Classification of non-coding RNAs.

Non-coding RNAs		Definition
**1. Structural non-coding RNAs**	tRNA and rRNA	A) The tRNA, 70–87 nucleotides in length, plays critical roles in translation by transferring amino acids to initialize and elongate peptides (38). B) In eukaryotes the cytoplasmic ribosomal RNAs (rRNAs) are the 5S (~120 nucleotides), the 5.8S (~150 nucleotides), the 18S (~1800 nucleotides) and the 28S (~4000 to 5000 nucleotides). Play critical role in protein synthesis (39).
**2. Regulatory non-coding RNA**	**2.1. small non-coding RNAs**: microRNA (miRNA), Piwi-interacting RNA (piRNA), Small interfering RNA (siRNA), Centromere repeat associated small interacting RNA (crasiRNAs), telomere-specific small RNA (telsRNAs) (40).	Size between 20–50 nucleotides, play critical roles in multiple regulatory processes, including transcription, post-transcription, and translation. miRNAs: are small non-coding RNAs that span between 18-24 nucleotides. miRNAs regulate gene expression on a post-transcriptional level. miRNAs modulate and orchestrate cellular pathways, including cell growth, autophagy, apoptosis, autophagy, and migration pathway (18, 41).
	**2.2. medium non-coding RNA**: small nucleolar RNA (snoRNA), transcription initiation RNA (tiRNA), small nuclear RNA (snRNA), small cytoplasmic RNA (scRNA), PROMPTs (40).	Size between 50–200 nucleotides (40).
	**2.3. long non-coding RNAs**: **2.3.1. Biogenesis**: Intronic RNA, Enhancer RNA, Promoter RNA, Antisense RNA, Sense RNA, Intergenic RNA, Bidirectional RNA (40). **2.3.2. Structure**: a) Linear lncRNA, b) Circular lncRNA, c) Long intergenic noncoding RNAs (lincRNA), enhancer-derived RNAs (eRNAs), transcribed ultraconserved RNAs (TUCRNAs), Natural antisense transcript (NATs) (40). **2.3.3. Action**: cis-acting long non coding RNA (cis-lncRNA), competing endogenous RNA (ceRNA), trans-acting long non coding RNA (trans-lncRNA) (40).	Size greater than 200 nucleotides (40).

Alterations in the molecular mechanisms of cell death are a common feature of oral cancer, like other cancer types. These alterations enable malignant cells to survive intrinsic death signaling leading to the accumulation of genetic aberrations and helping them to cope with adverse conditions (42). Although pharmacological targeting of cell death pathways has been the subject of intensive efforts in recent decades with a dominant focus on targeting apoptosis, the identification of factors like ncRNAs that regulates death pathways can open novel avenues for intervention in oral cancer cells and the immune system. In this mini-review, we discuss the importance of ncRNAs in the regulation of two key cell death processes, apoptosis, and necroptosis, of oral cancer cells and delineate the role of ncRNAs in controlling the molecular networks of these forms of cell death during oral carcinogenesis.

## Oral Cancer

Oral cancer is a subcategory of head and neck cancers that initiates inside the mouth involving anterior two-thirds of the tongue, gingivae, mucosal lining of lips and cheeks, sublingual floor of the mouth, the hard palate and the small retromolar area (43, 44). Signs and symptoms associated with oral cancer include a lump or non-healing sore/ulcer present for more than 14 days, presence of soft red, white or speckled (red and white) patches in the mouth, difficulty in swallowing, chewing, speaking, jaw or tongue movements, malocclusion or ill-fitting dentures and sudden weight loss (45). Oral cancers are the 6th leading cancer by incidence in the world and 90% of these are histologically squamous cell carcinoma (46). The 5-year survival rate is <50% in advanced cases with women having a more favorable outcome (47). The prognosis of these patients is always reliant on age, lymph node involvement and primary tumor size and location (42, 48). The most common risk factors include the premalignant conditions, consumption of tobacco, betel nut, alcohol along with poor oral hygiene, UV radiations, Epstein Barr Virus (EBV) and Human Papilloma Virus (HPV) especially HPV 16 and 18 (49).

Accumulating evidence supports the idea that human microbiome are strongly associated with several tumor types. Oral squamous cell carcinoma(OSCC) is the most frequently studied oral malignancy and it is also the most prevalent head and neck cancer overally. However, there is debate considering the potent role of oral microbiome in the development of OSCC. A distinctive composition of oral microbiome associated with OSCC was not found in previous studies. Individual oral microbiome have the capability to enhance different tumor-promoting mutations in the pathogenesis of OSCC,however a direct casusal relationship has not been yet verified (50, 51). Fusobacterium nucleatum and Porphyromonas gingivalis, the two main oral microorganisms, have been shown to enhance tumorigenesis in mice. Infection with P. gingivalis was associated with tumors of the oro-digestive tract, enhanced invasiveness of oral cancer and increase proliferation of related stem cells (50). Previous studies have shown that periodontal inflammation may enhance inflammation in the gut *in vivo* through transferring oral pathobionts to the gut, which activates colonic mononuclear phagocytes inflammasome and promotes inflammation (52, 53). Moreover, periodontitis leads to formation of reactive Th17 cells against oral pathogens. These reactive Th17 cells then migrate to the gut. In the gut, these orally-based Th17 cells become activated by oral pathobionts translocated from the oral cavity and initiate colitis, mweanwhile the micribiota of the gut do not activate these cells (50). Whether inflammation of the gut may exert similar effect on the oral cavity environment and increases the severity of inflammatory reactions and head and neck cancers, has not been yet elucidated. There are few studies on the impact of the gut microbiome on the immune response to oral cavity cancer, but they may be worthwhile to pursue.

The extent of oral cancer spread is estimated by staging the cancer. The commonly used staging system for oral cancer is TNM system, where T (for tumor) defines the size of the primary tumor. It is further categorized from 1 to 4 on the basis of tumor size, a higher number indicates larger size. N (for lymph nodes) shows extend of cancer spread to lymph nodes in the vicinity of the organ. It is further categorized to N0 (no spread), N1, N2, or N3. The N1-N3 shows the number of lymph nodes involved alongside their location and size. M (for metastasis) describes cancer spread to other parts of the body *via* lymph or blood. It is further classified to M0 (no spread) and M1 (spread). Overall oral cancer staging is given as **Table 2** (42):

**Table 2:** TNM classification of carcinomas of the lip and oral cavity (54).

Stage	Explanation
**Primary Tumor**	
T0	No evidence of primary tumor
TIS	Carcinoma in situ
T1	Tumor size 2cm or smaller, 5mm deep or less
T2	Tumor ≤ 2cm, >5mm and ≥ 10mm depth of invasion or tumor> 2cm but ≤ 4cm and depth of invasion ≤ 10mm
T3	Larger than 4cm, or deeper than 10mm
T4a	Extrinsic muscle of the tongue removed, included extensive tumors with bilateral tongue involvement and/or DOI>20 mm.
T4b	Very advanced locally disease definition; tumor invades masticator space, pterygoid plates, skull base, and/or encases the internal carotid artery.
**Regional lymph nodes (N)**	
N0	The lymph nodes don’t contain cancer cells.
N1	Metastases to the single lymph node. The node is no larger than 3cm across. Node must be extranodal extension negative.
N2a	Metastases to multiple ipsilateral nodes, and the node is between 3cm and 6cm across. Nodes must be extranodal extension negative or single ipsilateral or node 3cm or smaller with extranodal extension.
N2b	Metastases to multiple ipsilateral nodes, and the node is between 3cm and 6cm across. The Nodes must be extranodal extension negative.
N2c	metastases to bilateral nodes or contralateral nodes none >6 cm. Nodes must be extranodal extension negative
N3	Metastases to nodes >6 cm
N3a	Metastases to nodes >6 cm but extranodal extension negative.
	single ipsilateral nodes in machine learning >3 cm in greatest dimension with extranodal extension or multiple ipsilateral, contralateral, or bilateral nodes, any with extranodal extension or single contralateral node 3cm or smaller and with extranodal extension.

In addition, classification of tumors of the oral cavity and mobile tongue based on WHO classification is summarized in ***below***:

**(A)** Malignant Surface Epithelial Tumors: the major modification in this group is the oropharynx exclusion. Different OSCC(oral squamous cell carcinoma) subtypes are adenosquamous carcinoma,spindle cell carcinoma, basaloid SCC, papillary SCC,carcinoma cuniculatum, verrucous carcinoma, acantholytic SCC, and lymphoepithelial SCC (55). **(B)** Oral Potentially Malignant Disorders and Oral Epithelial Dysplasia: this group has been renamed from combining sections entitled Epithelial precursor lesions and Proliferative verrucous leukoplakia and precancerous lesions. A recently described type of dysplasia which is positive for high-risk HPV with characteristic histology (HPV-associated oral dysplasia) was added to the 4th edition, however the clinical significance of this finding is not clearly known as malignant transformation risk is poorly determined. **(C)** Papillomas : Condyloma acuminatum, Squamous cell papilloma, Verruca vulgaris, and Multifocal epithelial hyperplasia. **(D)** Tumours of uncertain histogenesis: Congenital granular cell epulis, and Ectomesenchymal chondromyxoid tumour. **(E)** Soft tissue and neural tumors: Rhbadomyoma, Granular cell tumour, Haemangioma, Lymphangioma, Kaposi sarcoma, Schwannoma,Myofibroblastic sarcoma and neurofibroma. **(F)** Oral mucosal melanoma, **(G)** Salivary type tumors: Mucoepidermoid carcinoma and Pleomorphic adenoma, and **(H)** Haematolymphoid tumours: CD30-positive T-cell lymphoproliferative disorder, Langerhans cell histiocytosis, plasmablastic lymphoma and extramedullary myeloid sarcoma (55–57).

The stage of the disease usually determines the primary option for the treatment of oral cancer. The treatment options include surgical resection, chemotherapy, radiotherapy, immunotherapy alone or in combination. Despite favorable advancements in the conventional therapeutic modalities, many disadvantages still need to be addressed; surgical resection may lead to long-lasting disfigurements, multiple corrective surgeries usually cause considerable deformities that leaves patients in psycho-social stress and isolation, whereas radio- or chemo- therapies end up with significant toxicities or treatment resistance, all compromising the patients’ quality of life and well-being (58, 59). Also, locoregional relapse may occur after years of the treatment leading to recurrent growth of the cancers (60). The effectiveness of different therapeutic modalities is largely dependent on the mutational profile of tumors as genetic alterations confer new oncogenic potential to cancer cells. The precise targeting of these alterations together with treatment regimen modifications decreases therapeutic resistance and may result in countless lives being saved from potential morbidity and mortality (42).

## Programmed Cell Death and Cancer

Cancer, a complex genetic disease resulting from mutation of oncogenes or tumor suppressor genes, can be developed due to alteration of signaling pathways; it has been well known to have numerous links to PCD (61). Apoptosis (type I PCD) is the major type of cell death that occurs when DNA damage is irreparable. Two core pathways exist to induce apoptosis, the extrinsic – death receptor pathway and intrinsic – mitochondrial pathway (62, 63). The extrinsic pathway is triggered by binding of Fas (and other similar receptors such as tumor necrosis receptor 1 and its relatives) plasma‐membrane death receptor with its extracellular ligand, Fas‐L. When death stimuli occur, Fas‐L combines with Fas to form a death complex. The Fas/Fas‐L composite recruits death domain‐containing protein (FADD) and pro‐caspase‐8, aggregating to become the death‐inducing signaling complex (DISC). Consequently, the protein complex activates its pro‐caspase‐8, which proceeds to trigger pro‐caspase‐3, the penultimate enzyme for execution of the apoptotic process (9). The intrinsic pathway also leads to apoptosis but under the control of mitochondrial pro‐enzymes. In both cases if a cell becomes initiated by either extracellular stimuli or intracellular signals, outer mitochondrial membranes become permeable to internal cytochrome c, which is then released into the cytosol. Cytochrome c recruits Apaf–1 and pro‐caspase‐9 to compose the apoptosome, which downstream triggers a caspase 9/3 signaling cascade, culminating (as concludes the extrinsic pathway) in apoptosis (64). Accumulating evidence has shown that abnormal expression of some key regulatory factors may lead to cancer, indicating the intricate relationships between apoptosis and cancer.

Autophagy is a major, regulated, catabolic mechanism with many links to processes that occur in malignant cells, and highly regulated by some autophagy‐related genes (ATGs). It is a crucial mechanism that responds to either extra‐ or intracellular stress, and can result in cell survival under certain circumstances; however, over‐activation of autophagy may result in autophagic cell death (65). When analyzing relationships between autophagy and cancer, a common challenge is to determine whether autophagy protects cell survival or contributes to cell death. Autophagy is well known to be crucial for cell survival under extreme conditions, and degradation of intracellular macromolecules provides energy required for minimal cell functioning when nutrients are scarce. Consequently, autophagic activation can play a protective role in early stages of cancer progression. On the other hand, however, autophagy can perform as a tumor suppressor by activating pro‐autophagic genes and blocking anti‐autophagic genes in oncogenesis (65, 66). However, reminiscent of the Roman god Janus, autophagy can also play the reverse part – a pro‐tumor role in carcinogenesis – by regulating a number of pathways involving Beclin‐1, Bcl‐2, Class III and I PI3K, mTORC1/C2 and p53 (67).

## Biogenesis and Function of MicroRNAs and Their Roles in Programmed Cell Death of Oral Cancer

MicroRNAs (miRNAs) are small (20–23 nts) and non-coding RNA molecules that negatively influence gene expression by binding to mRNAs and thereby promoting degradation of the target mRNA or blocking its translation into protein (19, 68–79).

MiRNA biogenesis is initiated from the nucleus similar to other RNAs (80–83). These non-coding RNAs are primarily synthesized as primary transcripts or pri-miRNAs by RNA polymerase II (RNA pol II), which are processed by the RNAse III, Drosha into pre-miRNAs with long hairpin precursors of ∼70–100 nucleotides. After Drosha processing, pre-miRNAs are transferred for cytoplasmic maturation. The protein exportin 5 combines with the pre-miRNA and GTP-binding nuclear protein RAN GTP and forms a transport complex (84). Following movement through the nuclear pore complex, GTP is degraded and leads to disassembly of the complex and release of the pre-miRNA into the cytoplasm (85, 86). Pre-miRNAs are then degraded by Dicer in the cytoplasm to generate mature miRNAs, leading to the production of a double-stranded ∼22-nt product, made up of the the mature miRNA guide strand and miRNA* passenger strand. This mature miRNA is subsequently loaded into the RISC(RNA-induced silencing complex). Accumulating evidence has found that the passenger strand may be loaded into the RISC complex in certain thermodynamic conditions (87, 88). RISC complex can detect target mRNA based on base-pair complementarity and lead to cleavage of the mRNA and/or suppression of the mRNA.

In addition, two strands comprise the miRNA duplex, known as the suffix -3p or -5p (26). One of these strands, is typically discarded (the passenger strand; annotated *), while the oher strand plays part in guiding eventual target selection of mRNA(the guide strand). Strand selection is mainly dependent on the thermodynamic characteristics of the duplex; the strand that is more loosely bound to the 5′-end of the duplex is likely to function as the guide strand. Additional features of miRNA guide strands include an excess of a purines (A/G rich) at the 5′-end and a U-bias, while the passenger strands contain excess pyrimidines (U/C rich) and a C-bias at the 5′-end. Meanwhile, the guide strand can be modified through different processes, such as duplex post-transcriptional modification, single point mutation in the duplex and specific proteins related to the Ago2 in the RISC complex(protein activator of dsRNA-dependent protein kinase versus trans-activation response RNA-binding protein) (89–92). Consequently, both arms comprising a pre-miRNA hairpin can guide miRNAs (93) that are biologically functional (19).

MicroRNAs act as key regulators of a wide variety of cellular mechanisms and physiological processes, such as cell cycle progression, cell division, apoptosis and necroptosis (62, 62, 94–102). Recent studies have demonstrated that numerous miRNAs can strongly affect the expression of pro- and anti-apoptotic genes, oncogenes, ER stress- and/or necroptosis-related genes (20, 103). Aberrations involving miRNAs implicated in apoptosis or necroptosis could also impact physiological conditions and promote disease, including carcinogenesis and infection diseases (19, 104–106). Numerous studies have demonstrated that miRNAs usually target multiple mRNAs and they could act as so-called apopto-miRs, oncomiR or tumor suppressor miRNAs in different cancers. Apopto-miRs are miRNAs involved in apoptosis, whereas oncomiRs promote tumor development (107). Elucidating the roles of oncomiRs and apopto-miRs in particular types of cancers can aid in their utility as tumor biomarkers for diagnosis, prognosis and selecting the most suitable treatment option for cancer patients.

MiRNAs can be used as treatment options in two different strategies. One approach is mediated by a gain of function and acts to suppress proto-oncogenic miRNAs through administering antagonists of miRNAs, including such as antagomiRs, locked-nucleic acids (LNA) and anti-miRs. The second approach, is directed by replacing a tumor-suppressor miRNA analogue to aim a loss of function. Among these strategies, the inhibitory approach is more generally and theoretically acceptable as it is in harmony with short interfering RNAs(siRNA) and small molecule inhibitors, however, the replacement strategy provides a novel opportunity to identify the therapeutic potential for tumor suppressors (108). Mimics of miRNA are synthetic oligonucleotides of two strands processed into single-stranded miRNA to control target genes expression in target cells (109). Gene therapy has been used in the past to therapeutically recover tumor suppressor levels in tumor tissues; however, a practical application of this strategy is still awaiting. MiRNAs bring in new opportunities, as in contrast to tumor-suppressor proteins, miRNA mimics are significantly smaller and do not usually require entery to the target cells to become activated and can be administered by systemic routes and technologies frequently also used by siRNAs. As a result, the delivery barrier for miRNA mimics appears to be lower than it is for protein-encoding DNA. Moreover, numerous different observations encourage the strategy of miRNA replacement therapy: (i)The majority of differentially expressed miRNAs are decreased in tumor tissue compared to normal tissue, suggesting that miRNAs are more likely to act as tumor suppressors than oncogenes (108, 110).; and (ii) suppression of the processing of endogenous miRNA leads to an oncogenic transformation and accentuates tumor formation, indicating that the miRNA tumor-suppressive role dominates against its oncogenic role (111). Another benefit of miRNA mimics is that it has a similar sequence to the naturally occurring miRNA, and thus is considered to target the same mRNAs, which is also regulated by natural miRNAs. Non-specific unusual effects are not frequent as miRNA mimics are thought to act as the naturally occurring counterpart of interactions between miRNA-mRNA, which have evolved over a billion years. However, the main reason to identify the therapeutic potentials of miRNAs is dependent on the fact that several oncogenes and oncogenic processes commonly dys-regulated in cancer, can be regulated by a single miRNA (112).As a result, miRNAs function in harmony with our previous observations of cancer as a “pathway disease”, which may only be effectively treated with modifying several oncogenic pathways (108, 113).

The imbalance between proliferation and apoptosis is one of the most remarkable features of cancer cells. Therefore, investigating the regulatory mechanisms of proliferation and apoptosis is of great importance for cancer research. It has been well documented that resistance to apoptosis caused by several genetic aberrations may promote the uncontrolled growth, invasion, and metastasis of cancer cells (114). Apoptosis, as programmed cell death, is mainly mediated by two pathways: The mitochondrial pathway and the death receptor pathway (62, 115). It has been confirmed that the release of cytochrome c from the mitochondria into the cytosol may trigger the apoptosis *via* promoting the pro-apoptotic protein Bax expression, followed by the downstream activation of caspase-9 and caspase-3 (116, 117). Subsequently, PARP, as a substrate of caspase-3, is cleaved and activated to induce apoptosis (118). Recently, Cui et al. (119) investigated the regulation of miR-378 in the mitochondrial-mediated apoptotic signaling pathway of oral squamous carcinoma cells (OSCC). The results of their study showed that overexpression of miR-378 suppressed the proliferation and induced apoptosis of OSCC cells, while knockdown of miR-378-3p/5p led to the opposite results. Besides, miR-378 overexpression promoted the release of cytochrome c from the mitochondria into the cytosol, up-regulated the Bax/Bcl-2 ratio, and raised the cleaved caspase-9, cleaved caspase-3, and cleaved PARP levels in OSCC cells. Conversely, inhibition of miR-378-3p/5p expression obtained the opposite results. These results suggested that miR-378 participated in the dysregulation of proliferation and apoptosis in OSCC cells (119).

**Figure 1:**
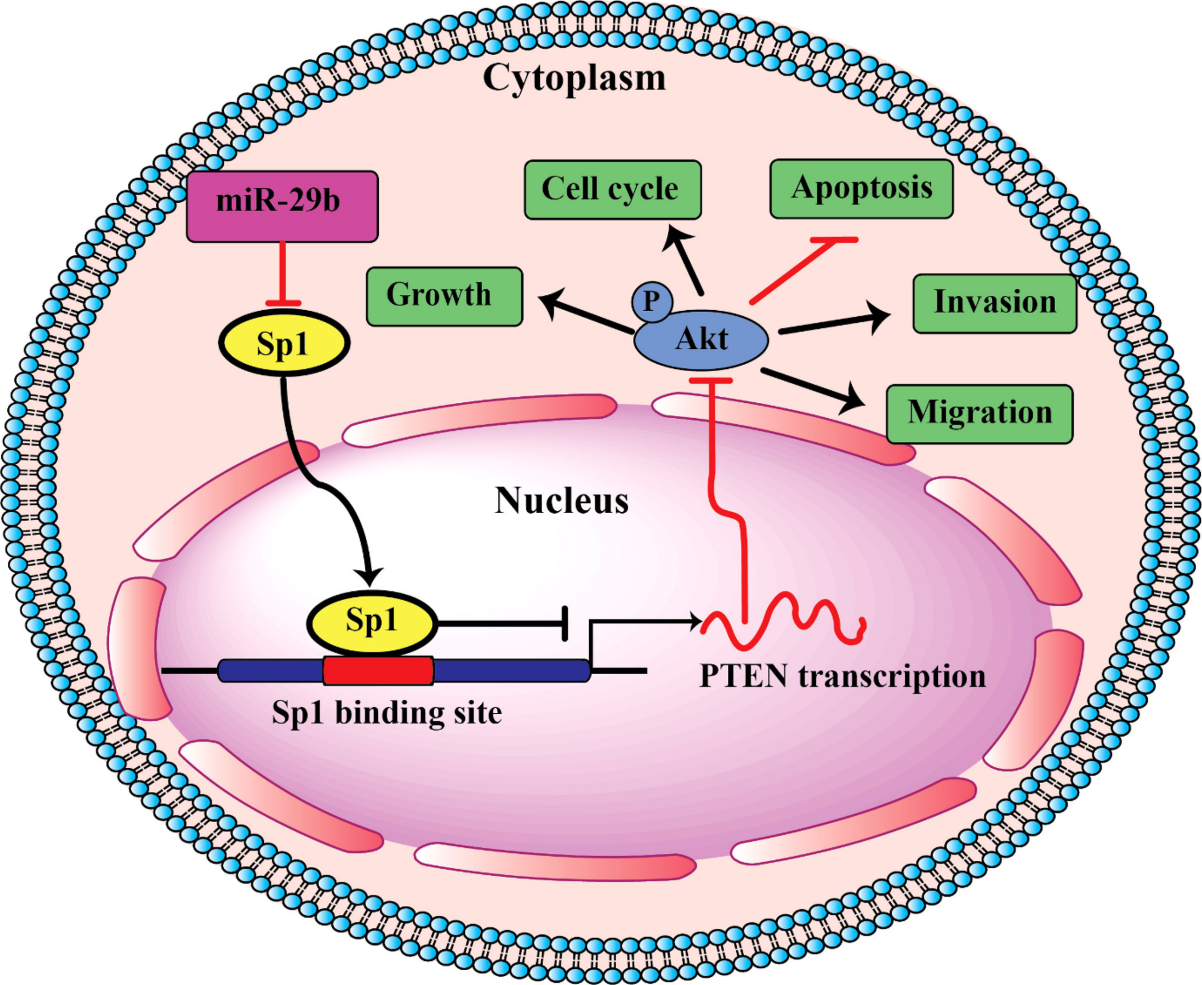
Schematic of pathways involved in the tumor-suppressor role of miR-29b in TSCC cells.

PI3K/AKT is an important signaling pathway, which regulates progression in numerous different types of tumors. The PI3K/AKT pathway is closely associated with apoptosis. This pathway controls cell proliferation, growth, translation, migration, and survival, and overactivation of this signaling pathway is associated with poor prognosis (120, 121). Activated PI3K may activate downstream protein kinase AKT; Activated AKT is able to phosphorylate Bax, inactivate Bax, and inhibit Bax and Bcl-2 to form dimers, thereby resulting in Bcl-2 dissociation and an anti-apoptotic effect (122, 123). Reportedly, miRNAs by regulating the PI3K/AKT signaling pathway can lead to modulation of oral cancer cell apoptosis. For example, miR-21-5p targeting PDCD4 suppresses apoptosis *via* regulating the PI3K/AKT/FOXO1 signaling pathway in tongue squamous cell carcinoma (TSCC). Also, inhibition of miR21-5p suppressed the PI3K/AKT/FOXO1 signaling pathway, suggesting that inhibiting the activation of the PI3K/AKT/FOXO1 signaling pathway may be a potential strategy for the treatment of TSCC (124). In addition, miR-378 overexpression was resulted in a decreased p-AKT level, whereas miR-378-3p/5p silencing could raise the p-AKT level in OSCC cells. Therefore, the PI3K/AKT signaling pathway was involved in the regulatory mechanisms of miR378 in OSCC (119). In addition, as shown in **Figure 1**, miR-29b suppresses proliferation, migration, and induces apoptosis of tongue squamous cell carcinoma through PTEN–AKT signaling pathway by targeting Sp1.

MiR-21 is commonly upregulated in toungue squamous cell carcinoma and carries an unfavorable prognosis by suppressing cellular apoptosis.On the other hand, its reduced expression was found to be associated with resistance to chemotherapy (125). In TSCC, high expression of miR-21 was associated with reduced expression of PTEN(phosphatase tensin homologue) and TPM1(tropomyosin1) (126). Several other important tumor suppressor and oncogenic targets were detected for miR-21 in tumors of the head and neck, including Ras, HNRPK, reversion inducing cysteine rich protein with kazal motifs (RECK)andprogrammed cell death 4 (PDCD4) (125). Furthermore, Zhang et al. (127), found that miR-21 can affect generation of ROS (reactive oxygen species) through direct mitigating the activity of SOD3 (superoxide dismutase family member 3), and through indirect attenuating the production of the pro-inflammatory mediator, tumor necrosis factor (TNF)-α, thus decreasing levels of SOD2 to facilitate carcinogenesis. Similarly, high expression of miR-2463 and miR-18462 is associated with decreased cellular apoptosis and augmented cellular proliferation (128). Upregulation of miR-184 exerts anti-apoptotic effects by targeting c-Myc and the apoptosis-related gene, TNFAIP2 (129, 130). Another study suggests that ectopic production of miR-184 inhibits the Akt pathway, which is linked to increased cell apoptosis and death, explaining the effects of miR-184 on epithelial cells and cancer cell lines *via* Akt signaling (131). Highly expressed miR-24 in oral squamous cell carcinoma appears to directly target DND1(RNA binding protein dead end 1), which leads to resistance to apoptosis.

p27Kip1 is a cyclin-dependent kinase inhibitor that regulates the progression of cells from G1 into the S phase in a cell cycle. p27Kip1 knockdown both inhibited G1 phase arrest and enhanced ER stress-induced cell apoptosis (132). Thus, deregulation of p27Kip1 has been associated with disease progression and an unfavorable outcome in several malignancies (133). Kudo et al. (134) demonstrated that Skp2 siRNA apparently inhibited p27Kip1 protein degradation, thus restraining oral cancer cell proliferation. It indicated that p27Kip1 upregulation played a role in weakening the malignancy of oral cancer cells (134). Fu et al. (135) exhibited that miR-155 targeted p27Kip1 gene and suppression of miR-155 lead to upregulated p27Kip1 level, reduced cell proliferation, and blocked cell cycle, which was similar to Kudo (134). In addition, Fu and colleagues (135) observed that antagomiR-155 transfection upregulated the CDC73 level in oral cancer cell line KB, thus promoting cell apoptosis and weakening the tumorigenicity in nude mice. This study revealed the role of miR-155 upregulation in reducing p27Kip1 expression and OSCC oncogenesis (135). As result, miR-155 regulates OSCC cells (Tca8113 cell) proliferation, cycle, and apoptosis *via* regulating p27Kip1.

Bmi-1, a polycomb ring finger oncogene, is highly expressed in multiple cancer cells and is involved in cancer cell proliferation, invasion, and apoptosis (136). Bmi-1 was first shown to collaborate with c-MYC in tumorigenesis in mice by suppressing the INK4A locus, which encodes (both p16Ink4a and p14ARF), two proteins that function to suppress cell proliferation and promote apoptosis (137). Recently, several studies have shown evidence for miRNA-mediated regulation of Bmi-1. miR-128a increases intracellular reactive oxygen species levels by targeting Bmi-1, which inhibits the growth of medulloblastoma cells (138) and miR-218 inhibits cell proliferation and cell cycle progression and promotes apoptosis by downregulating Bmi-1 in colorectal cancer cells (139). KIM et al. (140) suggested that the miR-203-induced apoptosis in YD-38 oral cancer cells was related to the suppression of Bmi-1. The result of this study demonstrated that miR-203 expression was significantly down-regulated in YD-38 cells compared to expression levels in normal human oral keratinocytes. miR-203 can decrease the viability of YD-38 cells in a time- and dose-dependent manner. In addition, over-expression of miR-203 significantly increased not only DNA segmentation but also the apoptotic population of YD-38 cells. These results indicate that miR-203 overexpression induces apoptosis in YD-38 cells. In addition, they found that both mRNA and protein levels of Bmi-1 were significantly reduced in YD-38 cells transfected with miR-203. These results indicate that Bmi-1 is a target gene of miR-203. miR-203 induces apoptosis in YD-38 cells by directly targeting Bmi-1, which suggests its possible application as an anti-cancer therapeutic. Although Bmi-1 is an important target of miR-203-induced apoptosis in YD-38 human oral cancer cells, they also suggested that miR-203 might regulate other genes (140).

Transforming growth factor (TGF)-β1 is known to regulate various cellular responses, including apoptosis, metastasis, cell proliferation, and differentiation (141). In the TGF-β1 pathway, Smad2 and Smad3 are receptor-regulated effector proteins, and TGF-β induces apoptosis through Smad-mediated expression of death-associated protein kinase (141). Recently, Huang and colleagues (142) investigated the role and mechanism of miR−18a-5p in OSCC and it was revealed that miR−18a-5p was highly expressed in SCC9 cells compared with normal cells. Then the effect of miR−18a-5p upregulation and miR−18a-5p downregulation was investigated on SCC9 cell viability, migration, invasion and apoptosis. They found that overexpression of miR−18a-5p could promote SCC9 cell viability, migration, invasion and inhibit cell apoptosis. While miR−18a-5p downregulation inhibited SCC9 cell viability, migration, invasion and induced cell apoptosis. These results indicated that miR−18a-5p acts as an oncogene to participate in OSCC progression and tumorigenesis by regulating the TGF-β1/Smad2 pathway (142). Also, several studies have shown miR-18a-5p was upregulated in several cancers such as (143, 144), hepatocellular carcinoma, prostate cancer and other cancers and can be act as oncomiR by inhibiting apoptosis of cancer cells (145–147). Also, in OSCC cells, miR−18a-5p acts as an oncogene by targeting tumor suppressor genes related to apoptosis.

HOX genes belong to a superfamily of homeobox genes that encodes transcription factors with important effects on modulating primary cellular processes, including cell recognition, growth, and differentiation, which was recently discovered to be regulated by miRNAs (148). Genes of the HOX family are highly expressed in leukemias, melanomas, and in breast, ovarian, cervical, esophageal, and prostate cancers (149–152). In addition, the aberrant expression of HOX genes was observed in OSCC (153). HOXB7, a member of the HOX family of homeodomain transcription factors, is a critical developmental regulator which influences the proliferation and survival of progenitor cells. However, its upregulation has been reported in several malignancies which contributed to tumorigenesis (154). Accumulating evidence has shown that HOXB7 was crucial to promoting cell proliferation and migration to participate in tumorigenesis and inhibit cell apoptosis (155, 156). Wang et al. (157) reported miR-376c-3p directly targeted HOXB7 and reduced the expression of HOXB7. They found that the expression level of MiR-376c-3p was down-regulated while HOXB7 was up-regulated in OSCC tissues and cells than the normal ones. Moreover, the overexpression level of miR-376c-3p suppressed proliferation, viability, migration, and invasion and induced G1/G0 arrest and cell apoptosis of SCC-25 cells. As result, they reported that miR-376c-3p induced cell apoptosis of OSCC *via* targeting HOXB7 and suggested it useful to replenish the expression of miR-376c-3p for the therapy of OSCC (157). miRNAs as regulators of apoptotic pathways are listed in **Table 3**.

**Table 3:** microRNA can regulate cell death pathways in oral cancer.

miRNAs	Expression	Cancer cell	Target of miRNA	Inhibition/Induction of Cell Death	Sample type	Note	Ref
miR-486-3p	Down	Oral squamous cell carcinoma (OSCC)	DDR1	Induction of apoptosis	Human (OSCC tissue, n= 40), *In vitro* (OEC-M1 and TW2.6 cells)	Overexpression of miR-486-3p can induces apoptosis by regulating DDR1 expression.	(1)
miR-4282	Down	OSCC	LIN28B	Induction of apoptosis	Human (OSCC tissue, n=72), *In vivo* (mice), *In vitro* (SCC15 and TSCCA cells)	miR‐4282 induces the apoptosis of OSCC cells through a decreased expression level of ZBTB2 *via* targeting LIN28B.	(2)
miR-17	–	Human tongue squamous cell carcinoma (TSCC)	–	Inhibition of apoptosis	*In vitro* (CAL-27 cells), *In vivo* (Xenograft tumor model (BALB/c nude mice))	MiR-17 suppresses the induction of cisplatin-induced apoptosis. Suppression of miR-17 causes induction apoptosis and autophagy by STAT3 signaling.	(3)
miR-17-5p	–	OSCC	–	–	*In vitro* (OC3 cells)	miR-17-5p increases (TRAIL R1, HIF-1α and cIAP-1) or decreases (p53, p21, FADD and TNF RI) apoptosis-related proteins in irradiated OC3 cell.	(4)
miR-27a	Up	TSCC	–	Inhibition of apoptosis	*In vitro* (Cal-27 cells and Clinical TSCC sample)	Decrease expression level of miR-27a lead to promote cell apoptosis in Cal-27 cells.	(5)
miR-145-5p	Down	TSCC and Oral cancer	–	Induction of apoptosis	Human (TSCC tissues), *In vitro* (SCC9 and Cal27 cell)	Upregulation of miR-145-5p levels cause promote cell apoptosis.	(6, 7)
miR-183	Up	TSCC	–	Inhibition of apoptosis	*In vitro* (SCC25 cell)	Downregulation of miR-183 levels lead to induction of apoptosis through decrease and increase expression level of casase-3 and BCL-xL, respectively.	(8)
miR-146a-5p	Up	OSCC	–	Inhibition of apoptosis	*In vitro* (SCC-9 cell)	The downregulation of miR-146a-5p levels leads to induces apoptosis in OSCC cells.	(9)
miR-149	Down	OSCC	–	Induction of apoptosis	*In vitro* (PECAPJ41 and HSC-4 cells)	Inhibition of miR-149 significantly suppresses apoptosis and autophagy.	(10)
miR-155	Up	Oral cancer cells	FoxO3a	Inhibition of apoptosis	*In vitro* (KB and KB/DDP cell line)	Inhibition of miR-155 significantly promotes apoptosis	(11)
miR-155	Up	OSCC	p27Kip1	Inhibition of apoptosis	Human (OSCC tissues, n=46), *In vitro* (Tca8113 cells)	Inhibition of miR-155 significantly promotes apoptosis od OSCC cells.	(12)
miR-378-3p/5p	Down	OSCC	KLK4	Induction of apoptosis	Human (OSCC tissues, n=30), *In vitro* (KB and Tca8113 cells)	miR-378-3p/5p promotes apoptosis of OSCC through targeting KLK4	(13)
miR-101-3p, miR-199b-5p	Down	Oral cancer	BICC1	Induction of apoptosis	Human (oral cancer tissues, n=62), *In vitro* (Tca8113, CAL-27, TSCCA, SCC-9) and *In vivo* (mice)	Overexpression of miR-199b-5p and miR-101-3p levels induces apoptosis by targeting BICC1 expression.	(14)
miR-214	Up	Oral cancer	MAPK/ERK signaling pathway	Inhibition of apoptosis	*In vitro* (HB cell lines)	miR-214 suppresses apoptosis by MAPK/ERK signaling pathway.	(15)
miR-214	Up	Oral cancer	RASSF5	Inhibition of apoptosis	*In vitro* (KB cell)	Downregulation of miR-214 expression lead to inhibition of apoptosis by targeting RASSF5.	(16)
miR-139	Down	Oral cancer	Akt signaling pathway	Induction of apoptosis	*In vitro* (Tca8113 cell)	Overexpression of miR-139 levels cause promote apoptosis through Akt signaling pathway.	(17)
miR-21−5p	Up	TSCC	PDCD4	Inhibition of apoptosis	Human (TSCC, n= 40), *In vitro* (Cal 27 and SCC9 cells)	miR-21-5p inhibits apoptosis by targeting PDCD4 and regulating the PI3K/AKT/FOXO1 signaling pathway.	(18)
miR-21	–	OSCC	–	Inhibition of apoptosis	*In vitro* (SCC15 and SCC25 cell)	miR-21 can inhibit apoptosis by up-regulating PTEN expression	(19)
miR-9	Down	OSCC	–	Induction of apoptosis	Human (OSCC, n= 21), *In vitro* (Tca8113 cells)	Overexpression of miR-9 levels cause induction of apoptosis	(20)
miR-203	Down	Oral cancer	Bmi-1	Induction of apoptosis	*In vitro* (YD-38 cells)	Upregulation of miR-203 levels cause induction of apoptosis by targeting Bmi-1.	(21)
miR-203	Down	Oral cancer	SEMA6A	Induction of apoptosis	*In vitro* (YD-38 cells)	miR-203 promotes apoptosis by targeting SEMA6A.	(22)
miR-4513	Up	OSCC	CXCL17	Inhibition of apoptosis	*In vitro* (Tca8113 and CAL-27)	Downregulation of miR-4513 levels promotes apoptosis.	(23)
miR-376c-3p	Down	OSCC	HOXB7	Induction of apoptosis	Human (OSCC, n= 49), *In vitro* (SCC-25 cells)	Overexpression of miR-376c-3p induces the apoptosis of SCC-25 cells by targeting HOXB7.	(24)
miR-375	Down	OSCC	IGF-1R	Induction of apoptosis	Human (OSCC, n= 44), *In vitro* (SCC-4)	miRNA-375 enhances apoptosis by targeting IGF-1R.	(25)
miR-124	–	OSCC	–	Induction of apoptosis	*In vitro* (KB and SCC9 cell)	Icaritin promotes mitochondrial apoptosis *via* increasing expression level of miR-124.	(26)
miR-26a	–	Oral cancer cells	Mcl-1	Induction of apoptosis	*In vitro* (KB cell)	Metformin promotes apoptosis through decreasing Mcl-1 expression by miR-26a.	(27)
miR−221/222	Up	OSCC	PTEN	Inhibition of apoptosis	*In vitro* (CAL27 and HSC6 cell)	miR−221/222 inhibits apoptosis by targeting PTEN.	(25)
miR-448	Up	OSCC	MPPED2	Inhibition of apoptosis	Human (OSCC, n= 15), *In vitro* (Cal−27 and Scc−9)	miR−448 inhibits apoptosis by targeting MPPED2.	(28)
miR-29a	Down	OSCC	MMP-2	Induction of apoptosis	Human (OSCC, n= 15), *In vitro* (SCC-4)	miR−29a induces apoptosis by targeting MMP-2.	(29)
miR-29b	Down	TSCC	Sp1	Induction of apoptosis	Human (OSCC, n= 40), *In vitro* (CAL27 cells)	Upregulation of miR-29b level cause promotes apoptosis in TSCC cells by regulating PTEN–AKT signaling pathway	(30)
miR-29a	Down	TSCC	Wnt/β-catenin pathway	Induction of apoptosis	Human (OSCC, n= 103), *In vitro* (HOK, SCC-25 cells)	Overexpression of miR−29a induces apoptosis by regulating Wnt/β-catenin pathway.	(31)
miR-9	Down	OSCC	CXCR4	Induction of apoptosis	*In vitro* (Tca8113 and SCC-9 cell)	Upregulation of miR-9 level cause promotes apoptosis in OSCC cells by targeting CXCR4	(27)
miR-218	–	OSCC	mTOR pathway	Induction of apoptosis	*In vitro* (OSCC cell line)	Overexpression of miR-218 reduces OSCC cell growth by caspase-mediated apoptosis.	(32)
miR-17-5p	Down	OSCC	p21	Induction of apoptosis	*In vitro* (OC3 cells)	Overexpression of miR-17-5p mediated by radiation treatment leads to induction of apoptosis by downregulation of p21 levels.	(33)
miR-1179	Up	Oral cancer cells	–	Inhibition of autophagy	*In vitro* (SCC-4, SCC-9, SCC-15 and SCC-25)	Inhibition of miR-1179 induces autophagy by regulation of MEK/ERK and PI3K/AKT signaling pathways.	(29)
miR-617	Down	OSCC	SERPINE1	Induction of apoptosis	*In vitro* (SCC-090 and PE/CA-PJ41)	Overexpression of miR-617 induces apoptosis of OSCC cells by negative regulation of SERPINE1.	(34)
miR-133a	Down	OSCC	CTBP2	Induction of apoptosis	Human (OSCC, n= 40), *In vitro* (UT-SCC-15 and CAL-27)	Overexpression of miR-133a induces apoptosis of OSCC cells by the suppression of CTBP2.	(35)
miR-149-3p	Down	OSCC	AKT2	Induction of apoptosis	*In vitro* (Cal27 and SCC-9)	miR-149-3p combined with Fluorouracil shows an additive effect on the OSCC cells apoptosis through targeting AKT2.	(36)
miR-198	Down	OSCC	–	Induction of apoptosis	*In vivo* (mice), *In vitro* (Cal-27 and SCC-9 cells)	Overexpression of miR-198 induces the apoptosis of OSCC cells.	(37)
miR-487-3p	Down	OSCC	PPM1A	Induction of apoptosis	Human (OSCC, n= 20), *In vitro* (CAL-27 and TCA8113)	miR-487-3p induces apoptosis of OSCC cell by targeting PPM1A.	(38)
miR−196a	Up	OSCC	FOXO1	Inhibition of apoptosis	*In vitro* (SCC9 cell)	Decrease the expression level of miR-196a induces apoptosis of SCC9 cell *via* regulating FOXO1 expression.	(39)
miR-103a-3p	Up	OSCC	RCAN1	Inhibition of apoptosis	*In vitro* (TSCCA and TCA8113 cells)	Inhibition of miR-103a-3p induces the apoptosis of OSCC cells by targeting RCAN1.	(40)
miR-195	Down	OSCC	–	Induction of apoptosis	*In vitro* (OSCC-15/DDP cells)	Overexpression of miR-195 induces apoptosis of OSC-15/DDP cells by regulating MEK1.	(18)
miR-92b	Up	OSCC	–	Inhibition of apoptosis	*In vitro* (CAL-27)	Inhibition of miR-92b induces the apoptosis of OSCC cells.	(41)
miR-101	Down	OSCC	TGF-βR1	Inhibition of apoptosis	*In vitro* (SCC-9 and Tca8113)	miR-101 induces apoptosis of OSCC cells through targeting TGF-βR1.	(42)
miR-22	Down	OSCC	WNT1	Induction of apoptosis	Human (OSCC, n=50) *In vitro* (Tca8113 and SAS)	miR-22 promotes apoptosis of OSCC cells *via* targeting WNT1.	(43)
miR-18a-5p	Up	OSCC	TGF-β1/Smad2 pathway.	Inhibition of apoptosis	*In vitro* (SCC9 cell)	Downregulation of miR-18a-5p promotes apoptosis of SCC9 cell by regulation of TGF-β1/Smad2 pathway.	(44)
miR−543	Up	OSCC	–	Inhibition of apoptosis	*In vitro* (SCC9, SCC25 and CAL27 cells)	miR−543 inhibits apoptosis of OSCC cell.	(45)
miR−199a−5p	Down	OSCC	IKKβ/NF−κB pathway	Induction of apoptosis	Human (OSCC, n= 60), *In vitro* (Tca8113 and SCC−4 cells)	miR-199a-5p promoted apoptosis of OSCC cells through regulation of the NF−κB pathway *via* targeting IKKβ.	(46)
miR-16	Down	OSCC	AKT3 and BCL2L2	Induction of apoptosis	*In vitro* (SCC-25 and CAL-27 cells)	miR-16 can induce apoptosis of OSCC cells by decreasing AKT3 and BCL2L2 levels.	(47)
miR-186	Down	OSCC	SHP2	Induction of apoptosis	Human (OSCC tissue, n=14), *In vitro* (Tca8113 and SCC-25 cells)	Overexpression of miR-186 induces apoptosis of OSCC cells through ERK and AKT pathways by targeting SHP2.	(48)
miR-1-3p	Down	OSCC	DKK1	Induction of apoptosis	*In vitro* (SCC-4 cells)	miR-1-3p promotes apoptosis of SCC-4 cells by regulating DKK1.	(49)
miR-626	Up	OSCC	RASSF4	Inhibition of apoptosis	Human (OSCC tissue, n=94), *In vitro* (f Ca9-22 and HSC2 cells)	miR-626 can inhibit apoptosis of OSCC cells by targeting RASSF4.	(50)

Human Herpesviruses (HHV), which are a group of large enveloped DNA viruses, have been found in several oral inflammatory diseases, indicating their role in progression of the disease. However, the viral components that cause oral illness are unknown. Interestingly, HHV also leads to expression of numerous miRNAs, known as viral miRNAs, which may be significantly involved in regulating pathogen-host interaction by regulating both the viral life cycle and host biological pathways. Viral miRNAs(v-miRs) have been detected in diseaded biopsies of the oral tissue and show their immunoregulatory roles. Accumulation of v-miRs in tumors of the oral cavity was observed in several studies (158, 159). For instance, nasopharyngeal carcinoma (NPC) due to EBV is a well-known oral cancer. Higher levels of seven EBV miRNAs were found in tissues from NPC and non-cancerous patients, with ebv-miR-BART7-3p being the most significantly enhanced (160). Ectopic expression of ebv-miR-BART7-3p in EBV-negative NPC cells causes a significant rise in metastases in mice, indicating that viral miRNAs play a role in tumor growth. Downregulation of PTEN induced by ebv-miR-BART7-3p, causes accumulation of the β-catenin and Snail.This leads to promoted EMT (epithelial-to-mesenchymal transition) and progression. An elevated EBV BART cluster miRNAs was also found in tissue samples of nasopharyngeal carcinoma (161). Additionally, ebv-miR-BART1 expression is significantly associated with distinct pathological features. In terms of mechanism of action, ebv-miR-BART1 (similar to miR-BART-7-3p) leads to direct downregulation of the PTEN levels and increased level of phosphorylated FAK,AKT, Shc,130Cas and ERK1/2. Accordingly, numerous miRNAs in herpes viruses cause synergistic regulation of the same gene/pathway to modulate pathological results. High expression of ebv-miR-BART7 was associated with resistance to treatment with cisplatin and increased cellular proliferation, migration and invasion *in vitro*. Using viral miRNAs as new markers of diagnosis in the progression of the disease course and treatment target in conjunction with pre-existing therapeutic modalities may bring a reliable and potent strategy for tumors and inflammatory diseases of the oral tract (158).

It has been shown that miRNAs may be applied as potent biomarkers in oral tumors. For instance, Maclellan et al. (162) explored the level of expression of different circulating miRNAs in patients with advanced oral lesions (high risk lesions is identified as oral cancer or carcinoma in situ). Their study found that 15 miRNAs were overexpressed and 5 miRNAs were prominently lowly expressed based on the clinical status of the disease.Therefore, their study confirmed that five miRNAs (miR-29a,miR-16, miR-223,let-7b and miR-338-3p)provide diagnostic potential as non-invasive markers for high-grade oral lesions or oral cancers (162) and that serum miRNA profiles combined with additional screening techniques may significantly enhance the sensitivity for detecting oral cancer.

## Biogenesis and Function of lncRNAs and LncRNAs-Mediated Programmed Cell Death of Oral Cancer

LncRNA is a special RNA type defined as transcripts over 200 nucleotides long, but is not translated into proteins (28, 70, 163–166). LncRNAs are numerous and abundant, and were originally considered to be biologically non-functional transcriptional noise.

In previous years, achievements in sequencing and analysis of genome facilitated the discovery of multiple lncRNAs and biogenesis of lncRNAs became unraveled (167). The majority of the lncRNAs are transcribed from exonic, intergenic and distal protein-encoding loci of the genome through the function of the enzyme RNA polymerase II, and only a tiny portion of these non-coding RNAs are generated by the RNA polymerase III (Pol III) complex or the single-polypeptide nuclear RNA polymerase IV complex. Then the pre-mature lncRNA becomes polyadenylated at the 3`-end and capped with methyl-guanosine at the 5′-end (168). It is usually modified by alternative splicing necessary to produce protein diversity (169). Alternative splicing can be categorized in three ways. Firstly, lncRNAs make interaction with certain splicing factors and then generate RNA-RNA duplexes with molecules of pre-mRNAs, and eventually, they affect the remodeling of chromatins, and thus accomplishing target genes splicing process (170). For instance, LINC-HELLP, a 205 kb-lncRNA, is found to be involved in regulating splicing and the pathogenesis of HELLP syndrome related to pregnancy.The mass spectrometry and purification experiments found that components of splicing such as the splicing-related factors Y-Box Binding Protein 1 (YBX1), Poly(RC) Binding Proteins 1 and 2 in addition to the the ribosomal machinery,can detect this lncRNA. The exact mechanism of regulating this splicing process by this lncRNA is poorly understood,however, it was shown that some protein (5′-end up to the middle) of the LINC-HELLP transcript loses its potential to make interactions with its protein partners due to the presence of mutation in patients with HELLP. On the contrary, mutations at the far 3′-end enhances the binding potential (170). Antisense, ‘as-Oct4-pg5’, and brain-associated’BC200’ are examples of functional lncRNAs that are not polyadenylated (171, 172).Ovrall, genes encoding lncRNAs have their unique promoters and DNA motifs and transcription factors(TFs) (173).

Epigenetic modification plays a major role in biogenesis of lncRNAs. Methylation of histone has a key role in regulating transcription. Methylation of histone H3 lysine 4 (H3K4) represents activation of transcription, while tri-methylated H3K27 symbolizes gene silencing. Several lncRNAs such as XIST, HOTTIP and FIRRE, etc. play role in activation of transcriptional genes and organizing 3D nuclear architecture (173). On the contrary, lncRNA decoys such as lncRNA-DNA triplex or Alu transcripts can suppress regulation of the transcription by binding to RNA pol II (174). Different transcription factors may bind to lncRNAs and produce a nascent transcript that eventually alters processing of the mRNA molecule through alternative splicing. Binding of the lncRNAs to mRNA can enhance or suppress protein translation or can facilitate mRNA decay (175). Small RNA deep sequencing(Srna-Seq) experiments have found that lncRNAs have also the potential to encode small functional RNAs (176). Mature lncRNAs are also present in the cell cytoplasm and/or nucleus (177). Although the cytoplasmic lncRNAs do not go under translation, small peptides were detected that were produced from lncRNAs due to their association with ribosomes (178). According to some studies, pseudogenes transcriptionally active may also generate these molecules or they can be formed through transcription of the promoter or intergenic loci (179).

Because of enhanced technologies for lncRNA functioning, structure, and interacting partners, our present understanding of lncRNA is more thorough. LncRNAs have major role in maintaining pluripotency of stem cells, differentiation and related human disorders caused by their dysfunction, and recently identified lncRNAs were shown to maintain pluripotency of stem cells and regulate cellular lineage differentiation. Importantly,the structure and location of the lncRNAs appears to forecast their function. LncRNAs can regulate reprogramming of somatic cells and differentiation of stem cells, thus their manipulation allows more efficient strategies for stem cell differentiation nd reprogramming of somatic cells (180). Accordingly, lncRNAs regulate different stages of human or mice reprogramming through several exogenous factors. lncRNA-Xist is involved in the development of an epithelial phenotype during the early stages of conversion from somatic cells to reprogramming intermediates, whereas LNCrna-ladr86/91 is implicated in the metabolic transition from aerobic to anaerobic energy generation (181). lincRNA-p21 plays role in both the early and late phases(reactivation of the pluripotency gene network) in reprogramming by inhibiting cellular proliferation and activation of pluripotency genes, respectively. Likewise, lincRNA-RoR modulates the initial phases of reprogramming through inhibition of p53, which is negative regulator of cell survival, as well as the late phase of reprogramming *via* sponging for miR-145, targeting several pluripotency gene transcripts. lincRNA-1526 and lincRNA-1463 are associated with inverse regulation of the activation of the pluripotency genes in reprogramming, while lincRNA-1307 and ladr49/83 can positively regulate the pluripotency genes. LNCPRESS1, Snhg14, Gas5 and Peblr20 are positive regulators of the pluripotency genes in reprogramming and/or embryonic stem cells(ESCs) (182). Moreover, there are novel approaches to comprehend regulation of the lncRNAs. For instance, RAT-seq allows for the discovery of the genome-wide chromatin binding sites for each specific lncRNA (183, 184). More significant functions and fresh mechanistic insights into how lncRNAs influence biological processes and illnesses will be discovered using these methods. These new findings might potentially lead to the discovery of novel therapeutic targets and the development of new treatments for human diseases (185).

LncRNAs can be divided into three different categories based on their location in the genome: natural antisense transcripts (NATs), long gene non-coding RNAs (lincRNAs) and intron lncRNAs. Upon increasing the binding of UCHL1 mRNA to the polymer, antisense lncRNA UCHL1 enhances the UCHL1 mRNA translation. On the other hand, other lncRNAs, such as lincRNA-p21, inhibit the translation of their target mRNAs after binding to them (186). SINEUPs comprises a novel and functional family of synthetic and natural antisense lncRNAs that can promote target mRNAs translation without producing any effect on the circulating level of mRNAs (187). This lncRNA is comprised of an SINE element (effector domain) embedded into its structure and a binding domain exerting target specificity (188). AS-Uchl1comprises a classic example of the natural SINEUPs. Carrieri et al. observed that AS-Uchl1 had the potential to enhance the translation of sense Uchl1 mRNA in dopaminergic neuronal cells in mice by promoting association of Uchl1 mRNA to heavy polysomes. This process is dependent on the AS-Uchl1 inverted SINEB2 element, which does not affect the level of Uchl1 mRNA (187). According to their other study, HNRNPK and PTBP1 functioned as RNA binding proteins to interact with SINEUP, and thus contributing to subcellular distribution of SINEUP RNA and assembly of initiation complexes of translation (189). Synthetic SINEUPs were found to be the primary measurable tool to enhance the expression of a specific target gene. For example, SINEUP-cox7B transcribed *in vitro* is a synthetic SINEUP produced against endogenous cox7B mRNA, which can efficiently and specifically up-regulate the expression and translation of COX7B protein, leading to rescuing size of the brain and eye tissues in cox7B morphants (190). Collectively, SINEUPs contain significant treatment potential that can be used in different diseases due to inadequate protein production.

Although lncRNA is not translated into protein, it is still considered a functional molecule that regulates gene expression at multiple levels, including the chromatin, transcriptional, and post-transcriptional levels (191). Indeed, lncRNA is involved in cell differentiation, cell cycle regulation, stem cell pluripotency, and maintenance of various biological processes (192, 193). LncRNAs can affect different apoptotic pathways. For instance, lncRNA overexpression can reduce the expression of membrane surface receptors by affecting extrinsic apoptosis pathway (194). Autophagy not only promotes cell survival but also causes cell death in some conditions. LncRNA can activate autophagy by activating related enzymes (194). In addition, lncRNAs can protect tumor cells from necroptosis by inhibiting the expression of some related proteins (195). Some lncRNA types also act as competitive endogenous RNAs to prevent oxidation and inhibit ferroptosis (195). Increasing evidence shows that lncRNAs are closely related to PCD, and the relationship between lncRNA and PCD is associated with the occurrence of heart disease, cancer cell apoptosis, and cell survival (196). In this section, we summarize the role of lncRNAs in PCD to determine the relationship between lncRNAs and PCD of oral cancer cells.

The evasion of apoptosis has been classified as one of the hallmarks of cancer and is among the main causes of therapy failure. Most tumors are defective in the activation of apoptosis owing to the occurrence of genetic or epigenetic events that either inactivate the p53 pathway or provoke the aberrant expression of pro-survival BCL-2 family members including BCL-2, BCL-XL or MCL-1 (197). It has been shown that BCL-2 exerts a key role in tumorigenesis. In a mouse model of APC-loss-induced colorectal cancer, BCL-2-dependent impairment of apoptosis is required for carcinoma onset (198). Importantly, such unbalance in favor of the pro-survival side of the BCL-2 family confers resistance to radiation, chemotherapeutic agents, and many selective pathway inhibitors that induce apoptosis primarily through the activation of the mitochondrial death machinery (197). Multiple regulatory mechanisms targeting BCL-2 family members involve a different class of lncRNAs. Several lncRNAs have been reported to mediate escape from apoptosis through different mechanisms. For example, lncRNA HOTTIP (HOXA distal transcript antisense RNA) promotes BCL-2 expression and induces chemoresistance in small cell lung cancer by sponging miR-216a (199). In another study, knockdown of HOTTIP has been shown to induce apoptosis by increasing Bax expression and decreasing Bcl-2 expression in prostate cancer cells (200). Sun et al. revealed that HOTTIP acted as a competitive endogenous RNA for microRNA-216a, thus preventing Bcl-2 from binding microRNA-216a in lung cancer cells (199). Recently, Mu et al. (201) reported that lncRNA HOTTIP knockdown lead to restrained the cell proliferation and arrested the cell cycle at G1 phase in human TSCC cell lines (TSCCA and TCA8113 cells). Furthermore, the expression levels of cyclins B, D1, and E were downregulated in HOTTIP-silenced cells. Also, HOTTIP silencing suppressed the growth of xenograft tumors. Besides, they found that the silencing of HOTTIP cause triggered apoptosis in TSCCA and TCA8113 cells and altered the expression of a group of apoptosis-related molecules: downregulated Bcl-2, upregulated Bax, and enhanced the cleavage of caspase 3 and PARP. Knockdown of HOTTIP also suppressed the migration, invasion, and epithelial–mesenchymal transition (EMT) of both TSCCA and TCA8113 cell lines. Altogether, the results of their study suggested that HOTTIP plays a critical role in regulating cell proliferation, apoptosis, and tumorigenesis of TSCC cells and it may serve as a promising potential candidate for OTSCC therapy (201).

Disorder of cell proliferation, apoptosis, and metastasis is an important trigger of cancer occurrence and development. The PI3K/AKT signaling pathway is an important signaling pathway that regulates cell proliferation, apoptosis, and metastasis (202). Recent studies have shown that autophagy plays an important role in tumorigenesis (203). Moreover, mTOR, which is downstream of the PI3K/AKT signaling pathway, is a crucial negative regulator of autophagy (13). Studies on esophageal squamous cell carcinoma and hepatocellular carcinoma have shown that high expression of lncRNA-CASC9 activates the PI3K/AKT signaling pathway, promoting the proliferation, invasion, and metastasis of cancer cells (204, 205). Recently, Yang et al. (206) observed that increased CASC9 expression promotes OSCC cell proliferation. It is unclear whether the increased CASC9 expression in cancer cells regulates the expression of mTOR through the regulation of the PI3K/AKT signaling pathway to control autophagy. For this reason, they depleted lncRNA CASC9 in OSCC cells which led to the significantly decreased expression of p-AKT and p-mTOR, as well as increased autophagy. Also, CASC9 knockdown partially rescued the decreased p-mTOR expression and increased autophagy. Taken together, these findings demonstrated that CASC9, which is highly expressed in OSCC cells, inhibits autophagy by activating the AKT/mTOR pathway (206).

Autophagy plays an important role in the occurrence and development of tumors (207). There exists crosstalk between autophagy and apoptosis; autophagy can both inhibit and promote apoptosis to affect the occurrence and development of cancers (208). Due to the diversity of cells, conditions and stimulating factors, autophagy acts as a double-edged sword for apoptosis (209). For example, Zhao Z. et al. found that oxamate inhibits apoptosis by promoting autophagy to promote gastric cancer (210); in contrast, Yeh P.S. et al. found that honokiol can promote apoptosis by promoting autophagy to promote neuroblastoma (211). As mentioned, Yang et al. (206) found that autophagy and apoptosis were both increased in cells with silenced CASC9. However, apoptosis is significantly reduced in the OSCC cells cotreated with silenced lncRNA-CASC9 and the autophagy inhibitor, indicating that silencing CASC9 induced autophagy as a pro-death response. This study suggested that autophagy can regulate early apoptosis. Their study also found that CASC9 knockdown in OSCC cells increased and decreased the early apoptosis and P62 protein expression, respectively (206). It is shown that P62 can regulate both autophagy and apoptosis, and many studies reported that P62 mediates apoptosis primarily through regulating autophagy (212). It has been reported that P62 is incorporated into autophagosomes through binding to LC3 in autophagy-activated cells, and subsequently P62 is degraded by autophagy (213); thus, P62 protein expression decreases, and reduced P62 protein expression increases cell apoptosis. Overall, Yang et al. (206) supposed that CASC9 depletion in OSCC cells might promote the binding of P62 to LC3, and then P62 is incorporated into autophagosomes, resulting in the degradation of P62; subsequently, reduction of P62 may increase the autophagy-mediated apoptosis (206). However, the detailed mechanism remains to be further studied.

**Figure 2:**
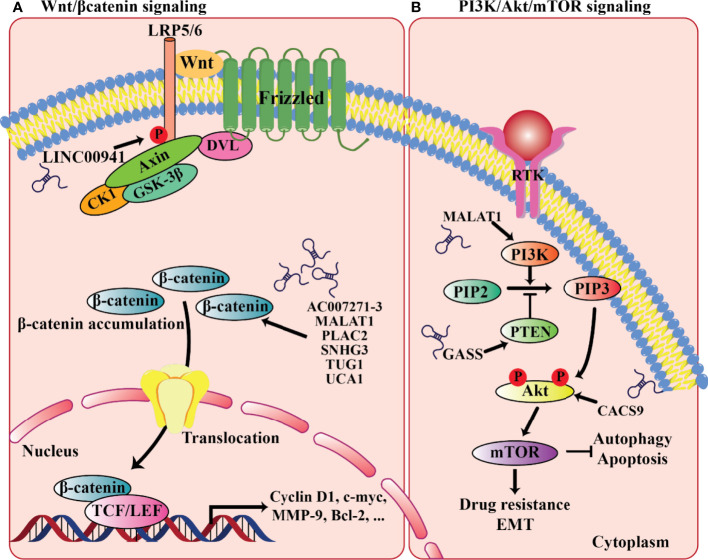
LncRNAs involved in the relevant signaling pathways implicated in oral cancer progression. **(A)** Wnt/β-catenin signaling. **(B)** PI3K/AKT/mTOR signaling.

Currently, for the treatment of OSCC, cisplatin (DDP) is the first-line chemotherapy agent, whereas acquired DDP resistance greatly diminishes drug efficacy and survival benefit. In DDP-resistant OSCC, Wang et al. reported the increased expression of lncRNA MALAT1. Specifically, by the activation of PI3K/AKT/mTOR signaling pathway, MALAT1 overexpression could lead to DDP resistance, whereas MALAT1 knockdown could effectively re-sensitize OSCC cells to DDP treatment, suggesting an instrumental role of MALAT1 in PI3K/AKT/mTOR signaling-associated DDP resistance development (214). Furthermore, PI3K/AKT/mTOR signaling is also known as a critical regulatory pathway of autophagy, a highly conserved catabolic process involving the lysosome-mediated degradation of intracytoplasmic components and participating in a variety of cellular biological activities (215). Autophagy may block or promote tumor survival, depending on the various tumor types and stages. Recently, in OSCC, another lncRNA CASC9 was found to promote cancer progression through suppressing autophagy-mediated cell apoptosis *via* inducing AKT phosphorylation and the subsequent activation of the AKT/mTOR pathway (206) (**Figure 2**). In contrast, the tumor-suppressive lncRNA GAS5 (growth-arrest-specific transcript 5) could suppress proliferation, migration, invasion, and epithelial-mesenchymal transition (EMT) in OSCC (216, 217). Mechanically, GAS5 has been reported to act as a miR-21 sponge in ovarian and cervical cancer. In OSCC, Zeng et al. observed that GAS5 most likely also functions in this way. By sequestering miR-21, GAS5 rescues the expression of PTEN, a negative regulator of PI3K signaling, from miR-21-mediated repression, and thereby inhibiting the PI3K/AKT pathway (216) (**Figure 2**). Taken together, accumulating reports have revealed the prominent involvement and implications of lncRNAs in regulating cellular proliferation, drug resistance, and apoptosis through targeting the PI3K/AKT/mTOR signaling cascade during oral carcinogenesis.

Autophagy suppresses tumor initiation and prevent s genomic instability. However, autophagy contributes to the progression of advanced cancer by rendering cancer cells resistant to therapeutic agent. Studies proved that the level of autophagy can be determined by monitoring its modulators, such as Beclin1, MAP1LC3B, mTOR, and P62 expressions (218, 219). Takamura et al. indicated that the deficiency of Beclin1/Atg6 could induce tumorigenesis of liver in mouse, and Atg5/Atg7 knockout mouse led to P62 gene deficiency and loss of autophagy. Abnormal accumulations of P62 inhibited NF -κB signing way and triggered inflammation, which promoted tumorigenesis. However, upon recovery of autophagy, inflammation was inhibited, thereby preventing tumorigenesis (220). Hu et al. reported that low expression of Beclin1 was related to poor differentiation, lymphatic metastasis, and TNM stag e and was a poor prognostic marker of OSCC (221). Increasing evidence s showed that autophagy can be regulated by HOTAIR (222). Yang L et al. found that in hepatocellular carcinoma, the overexpression of HOTAIR activate d autophagy by up -regulating ATG3 and ATG7, and the level of autophagy was inhibited when HOTAIR was knocked down (223). Luan W et al. reported that HOTAIR promoted the growth and metastasis of melanoma cells by competitively binding miR-152-3p and activating the downstream PI3k/Akt/mTOR signaling pathway (224). Wang et al. indicated that silencing of Beclin1 in OSCC promoted proliferation, inhibited apoptosis and enhanced chemosensitivity (225). However, whether HOTAIR could regulate autophagy was unclear. This researchers in another study found that HOTAIR interference could decrease the autophagosomes and the expression s of Beclin1, MAP1LC3B, ATG3 and ATG7 were down -regulated (226). Furthermore, it increased the expression of mTOR. So, HOTAIR could promote autophagy in OSCC cells. Besides, they observed that apoptosis rate accelerated in OSCC cells after HOTAIR was silenced, with increased expressions of bim, caspase 3, and caspase7 and decreased expressions of bcl - 2 and mcl -1 decreased. As result, HOTAIR acts as an oncogene in OSCC cells by accelerating autophagy and reducing apoptosis as well as silencing HOTAIR improves sensitivity to cisplatin for OSCC (226).

Maternally expressed 3 (MEG3), a long non-coding RNA, displays variety of biological functions, involved in diverse diseases. Researches have showed that MEG3 conferred endothelial cell aging and disturbed the regenerative angiogenesis (227). By negatively regulating notch pathway, MEG3 impaired the functional recovery after ischemic brain injury (228). The evidences indicated that MEG3 associate with vascular function pathological processes. MEG3 contributed to neurons apoptosis induced by hypoxia through miR-181b12/15-LOX axis (229), suggested that MEG3 may be infaust for neuroprotection. In the trophoblast cells of preeclampsia, level of MEG3 was down-expressed and resulted in abnormalities of apoptosis and migration. Although the functions of MEG3 were diversiform in nontumor disease, the tumor suppressor effect of MEG3 was concerned widely (230–232). In addition, recent studies have shown that the downregulation of MEG3 in lung cancer cells can activate the WNT pathway (230). The WNT/β-catenin signaling pathway, which regulates a variety of cellular processes, such as cell proliferation, apoptosis, and differentiation, is one of the classical pathways for signal transduction (233, 234). Liu and colleagues reported that lncRNA-MEG3 levels were negatively correlated with the WNT pathway. They reported also the expression level of MEG3 was significantly decreased in OSCC and overexpression of MEG3 inhibited the proliferation and metastasis of cancer cells and promoted apoptosis. Overall, they suggested that MEG3 inhibited OSCC cell proliferation, metastasis and promotes apoptosis by negatively regulating the WNT pathway (235). Recently, Tan et al. (236) reported lncRNA-MEG3 was downregulated in OSCC tissues, and the overexpression of MEG3 suppressed migration and promoted apoptosis in OSCC cell lines, while inhibition of MEG3 exhibited opposite effect. Also, they found that MEG3 could effectively sponge miR-548d-3p and decrease its expression level. Moreover, miR-548d-3p repressed the expression of SOCS5 and SOCS6 through binding their 3’UTR, thereby modulating the JAK–STAT signaling pathway and functioning as an oncogene in OSCC cells, as, overexpressed miR-548d-3p inhibits apoptosis by regulating the JAK–STAT Pathway. Importantly, overexpression of MEG3 enhanced the expression of SOCS5 and SOCS6 to regulate JAK–STAT pathway, whereas miR-548d3p overexpression decreased the effects of MEG3 on levels of SOCS5/SOCS6. Furthermore, upregulated expression of miR-548d-3p could abrogate the effect of MEG3 overexpression on migration and apoptosis in OSCC cell lines. In addition, the overexpression of MEG3 inhibited tumor migration and facilitated apoptosis *in vivo*. Together, the result of their study revealed that MEG3 could modulate JAK–STAT pathway *via* miR-548d3p/SOCS5/SOCS6 to suppresses migration and promote apoptosis in OSCC (236). Ferroptosis, an iron-dependent programmed cell death, can affect the prognosis of several tumors. A number of lncRNAs can alter the outcome of tumors by modulating the process of ferroptosis (237).In a study of Li et al. (238), the OSCC prognostic model was established on the basis of 8 frlncRNAs which have a prognostic role and expressed simultaneously with 25 mRNAs. Their study supported a prognostic model in patients with OSCC comprised of 8 different frlncRNAs, including MIAT,AC099850.3, STARD4-AS1,AC090246.1, AC021087.4, HOTARM1,ALMS1-IT1and AL512274.1., which can be used as a prognostic indicator and immune evaluation in OSCC patients and can be subsequently be used as promising therapeutic targets in these patients (238). However, little evidence is present considering the regulatory functions of ncRNAs in oral cancer by ferroptosis. The lncRNAs which regulate cell death pathways are listed in **Table 4**.

**Table 4:** lncRNAs can regulate cell death pathways in oral cancer.

lncRNAs	Expression	Cancer cell	Target of miRNA	Inhibition/Induction of Cell Death	Sample type	Note	Ref
LncRNA PANDAR	Up	OSCC	PIM1	Inhibition of apoptosis	*In vitro* (SAS cell line)	PANDAR inhibits apoptosis by overexpression of PIM1 through binding to SRSF7.	(51)
lncRNA GACAT1	Up	OSCC	miR-149	Inhibition of apoptosis and autophagy	Human (OSCC, n= 20), *In vitro* (PECAPJ41 and HSC-4 cells)	miR-149 induces apoptosis and in contrast, lncRNA GACAT1 inhibits apoptosis by sponging miR-149.	(10)
LncRNA PART1	Up	OSCC	FUS/EZH2 pathway	Inhibition of apoptosis	Human (OSCC, n= 36), *In vitro* (Tca-8113 and CAL27)	LncRNA PART1 inhibits apoptosis by binding to FUS and stabilizing EZH2 gene.	(52)
lncRNA PTSC3	Down	OSCC	–	Induction of apoptosis and autophagy	Human (OSCC, n=15), *In vitro* (SCC-1 and SCC-9 cells)	Overexpression of lncRNA PTSC3 induces apoptosis and autophagy.	(53)
LncRNA SCIRT	Down	OSCC	miR-221	Inhibition of apoptosis	*In vitro*	LncRNA SCIRT inhibits apoptosis through sponging miR-221 to upregulate lncRNA GAS5.	(54)
LncRNA LINC01207	Up	OSCC	miR-1301-3p	Inhibition of apoptosis and autophagy	Human (OSCC, n=30), *In vitro* (HSC-3 and HSC-4 cells)	LncRNA LINC01207 inhibits apoptosis and autophagy by regulating miR-1301-3p/LDHA axis.	(55)
LncRNA SNHG16	Up	OSCC	–	Inhibition of apoptosis	Huan (OSCC, n=50), *In vivo* (mice), *In vitro* (CAL27 and TCA8113)	LncRNA SNHG16 inhibits apoptosis.	(56)
lncRNA HOXA-AS2	Up	OSCC	EZH2	Inhibition of apoptosis	*In vitro* (Tca-8113 cells)	Downregulation of lncRNA HOXA-AS2 promotes apoptosis by regulating HOXA-AS2/EZH2 axis.	(57)
LncRNA HITTERS	Up	OSCC	MRE11-RAD50-NBS1 complex	Inhibition of apoptosis	Huan (primary OSCC, n=48), *In vitro* (SCC25 and CAL27)	lncRNA HITTERS can upregulate the expression level of proteins involved in DNA damage repair by binding to the MRE11-RAD50-NBS1 complex and as a result, attenuate endoplasmic reticulum stress-induced apoptosis.	(58)
lncRNA HOXA11-AS	Up	OSCC	miR-98-5p/YBX2 axis	Inhibition of apoptosis	*In vitro* (OSCC=15 and SCC-25 cells)	Downregulation of lncRNA HOXA11-AS promotes apoptosis by regulating miR-98-5p/YBX2 axis.	(59)
lncRNA DANCR	Up	OSCC	miR-216a-5p	Inhibition of apoptosis	Human (OSCC, n=86), *In vitro* (SCC15 and CAL-27)	Downregulation of DANCR induces apoptosis by targeting miR-216a-5p.	(60)
lncRNA PART1	Down	TSCC	miR-503-5p	Induction of apoptosis	Human (TSCC, n=40), *In vitro* (CAL-27 and SCC9 cells)	miR-503-5p prevent the effects of lncRNA-PART1 on the apoptosis, in return, overexpression of lncRNA-PART1 induces apoptosis by targeting miR-503-5p.	(61)
lncRNA PVT1	Up	OSCC	miR−150−5p/GLUT−1	Inhibition of apoptosis	Human (OSCC, n=70), *In vitro* (SCC-090, SCC-25 and CAL-27)	miR-150-5p can induce apoptosis, in return, lncRNA PVT1 inhibits apoptosis by regulating the miR-150-5p/GLUT-1 axis.	(62)
Exosomal lncRNA HEIH	Up	TSCC	miR−3169-5p	Inhibition of apoptosis	*In vitro* (SCC4/DDP cells)	Exosomal HEIH inhibits apoptosis by sponging miR−3169-5p and increasing HDGF levels.	(63)
LncRNA NEAT1	Up	OSCC	Notch signaling pathway	Inhibition of apoptosis	*In vitro* (TSCC1 cell)	LncRNA NEAT1 inhibits apoptosis by activating Notch signaling pathway.	(64)
lncRNA XIST	Up	OSCC	miR-27b-3p	Inhibition of apoptosis	Human (OSCC tissue), *In vitro*	lncRNA XIST inhibits cell apoptosis in OSCC by regulating miR-27b-3p.	(65)
lncRNA AC007271.3	Up	OSCC	Slug and Wnt/β-catenin signaling pathway	Inhibition of apoptosis	Human (OSCC, n= 97), *In vitro* (SCC9, SCC15, SCC25)	lncRNA AC007271.3 inhibits apoptosis in OSCC cells *via* regulating Slug	(66, 67)
LncRNA LINC00958	Up	OSCC	miR-185-5p/YWHAZ axis	Inhibition of apoptosis	*In vitro* (SCC 9 and CAL27)	LncRNA LINC00958 inhibits apoptosis through sponging miR-185-5p/YWHAZ axis	(68)
LncRNA LINC00958	Up	OSCC	Sirtuin1	Inhibition of apoptosis	*In vitro* (HSC4 cells)	LINC00958 increases anti-apoptosis protein Bcl-2 levels and decreases pro-apoptosis proteins Bax, c-casp-3. LINC00958 inhibits apoptosis of OSCC by targeting Sirtuin1.	(69)
lncRNA HOXA-AS2	Up	OSCC	–	Inhibition of apoptosis	*In vitro* (TCA-8113 cells)	Downregulation of lncRNA HOXA-AS2 induces apoptosis.	(70)
lncRNA SNHG20	Up	OSCC	miR-29a/DIXDC1/Wnt axis.	Inhibition of apoptosis	*In vitro* (SCC9 cells)	Downregulation of lncRNA SNHG20 promotes apoptosis through regulating the miR-29a/DIXDC1/Wnt axis.	(19)
lncRNA CASC9	Up	OSCC	AKT/mTOR pathway	Inhibition of apoptosis/autophagy	Human (OSCC, n=84), *In vitro* (SCC15 and CAL27)	lncRNA CASC9 inhibits autophagy-mediated cell apoptosis through the AKT/mTOR pathway.	(71)
lncRNA MEG3	down	OSCC	miR-548d-3p	Induction of apoptosis	*In vivo* (mice), *In vitro* (H157 and HSC-2 cells)	lncRNA MEG3 induces apoptosis through modulate JAK–STAT pathway by miR-548d-3p/SOCS5/SOCS6	(72)
lncRNA RP5-916L7.2	Up	TSCC	miR-328 and miR-939	Inhibition of apoptosis	Human (TSCC, n=30), *In vitro* (Tca-8113)	lncRNA RP5-916L7.2 suppresses cells apoptosis by targeting miR-939-5p and miR-328-5p.	(73)
lncRNA HOXA11-AS	Up	OSCC	miR-214-3p	Inhibition of apoptosis	Human (OSCC, n=31), *In vitro* (TSCCA and CAL-27)	LncRNA HOXA11-AS decreases apoptosis and caspase 3 activities by downregulation expression level of miR-214-3p.	(74)
ncRNA LEF1-AS1	Up	OSCC	–	Inhibition of apoptosis	Human (OSCC, n= 88), *In vitro* (SCC4 and SCC15 cells)	Downregulation LEF1-AS1 lead to induces apoptosis in OSCC cells.	(75)
lncRNA HULC	Up	OSCC	–	Inhibition of apoptosis	*In vitro* (SCC15 and SCC25)	Suppression of lncRNA HULC induces apoptosis in OSCC cells.	(76)
lncRNA CRNDE	Up	OSCC	–	Inhibition of apoptosis	Human (OSCC, n= 52), *In vitro* (CAL-27 and SCC-15 cells)	Suppression of lncRNA CRNDE induces the apoptosis of OSCC cells.	(77)
lncRNA KCNQ1OT1	Up	OSCC	miR-185-5p/Rab14 axis.	Inhibition of apoptosis	Human (OSCC, n= 60), *In vitro* (SCC15 and HSC-3 cells)	Suppression of lncRNA KCNQ1OT1 induces apoptosis by the regulation of miR-185-5p/Rab14 axis.	(78)
lncRNA GAS5	–	OSCC	miR-1297	Induction of apoptosis	*In vitro* (UM-SCC6 cells)	Overexpression of lncRNA GAS5 by propofol induces cell apoptosis. Besides, miR-1297 inhibits GSK3β expression. Upregulation of GSK3β levels by targeting miR-1297 through lncRNA GAS5 lead to enhances Mcl-1 degradation and induction of cell apoptosis.	(79)
LncRNA CASC2	Down	OSCC	miR-21	Induction of apoptosis	Human (OSCC, n=69), *In vitro* (Tca8113 and TSCCa)	LncRNA CASC2 promotes cell apoptosis by regulating of miR-21 expression.	(80)
LncRNA ENST00000 470447.1	Down	OSCC	–	Induction of apoptosis	*In vitro* (Tca-8113 cells)	ENST00000470447.1 promotes the apoptosis of Tca-8113 cells.	(81)
LncRNA LINC00961	Down	OSCC	PI3K/AKT signaling pathway	Induction of apoptosis	Human (OSCC, n=35), *In vitro* (OSC-4 and CAL-24)	LncRNA LINC00961 promotes apoptosis of OSCC cells by regulating the PI3K/AKT signaling pathway.	(82)
lncRNA ANRIL	Up	OSCC	TGF-β/Smad pathway	Inhibition of apoptosis	Human (OSCC, n=35), *In vitro* (OSC-4 and CAL-24)	lncRNA ANRIL inhibits apoptosis of OSCC cells by regulating TGF-β/Smad pathway.	(83)
LncRNA SNHG16	Up	OSCC	–	Inhibition of apoptosis	*In vitro* (CAL-27 and TSCCA cells)	LncRNA SNHG16 inhibits apoptosis of OSCC cells.	(84)
lncRNA XIST	Up	OSCC	miR-34a-5p	Inhibition of apoptosis	*In vitro* (Cal-27 and Tca-8113 cells)	Downregulation of lncRNA XIST promotes apoptosis of OSCC cells by regulating expression level of miR-34a-5p.	(85)
lncRNA LINC00662	Up	OSCC	–	Inhibition of apoptosis	Human (OSCC, n=61), *In vitro* (ISG15 cell)	Downregulation of lncRNA LINC00662 promotes apoptosis of OSCC cells.	(86)
lncRNA HOTAIR	Up	OSCC	–	Inhibition of apoptosis and induction of autophagy	*In vitro* (CAL-27)	lncRNA HOTAIR can act as an oncogene lncRNA in OSCC cells by decreasing apoptosis and promoting autophagy.	(87)
LncRNA HOTAIR	Up	OSCC	–	Inhibition of apoptosis	Human (OSCC, n= 76), *In vitro* (Tca8113 and TSCCA)	Downregulation of HOTAIR induces apoptosis of OSCC cells.	(88)
LncRNA HOTAIR	Up	OSCC	–	Inhibition of apoptosis	Human (OSCC, n= 45), *In vitro* (Tca8113)	Downregulation of HOTAIR induces apoptosis of OSCC cells.	(89)
lncRNA HOTAIR	–	OSCC	–	Inhibition of apoptosis	*In vitro* (Tca8113 cells)	DNA damage promotes overexpression of lncRNA HOTAIR mRNA and it inhibits apoptosis of Tca8113 cells.	(90)
lncRNA LACAT1	Up	OSCC	miR-4301	Inhibition of apoptosis	*In vitro* (Cal-27 and Tca-8113 cells)	LACAT1 suppresses apoptosis of OSCC cells by targeting miR-4301.	(91)
lncRNA C5orf66−AS1	Down	OSCC	–	Induction of apoptosis	Human (OSCC, n= 30), *In vitro* (SCC9 cells)	Overexpression of lncRNA C5orf66−AS1 promotes apoptosis of OSCC cells.	(92)
lncRNA HOTTIP	Up	TSCC	–	Inhibition of apoptosis	Human (TSCC, n= 30), *In vitro* (TSCCA and TCA8113 cells)	Downregulation of lncRNA HOTTIP promotes apoptosis of TSCC cell by regulation of apoptosis-related molecules	(93)
lncRNA NEAT1	Up	OSCC	miR-365	Inhibition of apoptosis	Human (OSCC, n= 30), *In vitro* (SCC9 cells)	Downregulation of lncRNA NEAT1 promotes apoptosis in OSCC cells by regulating miR−365.	(94)
lncRNA KCNQ1OT1	Up	Tongue cancer	EGFR	Inhibition of apoptosis	Human (tongue cancer tissues, n= 50), *In vitro* (UM1 and CAL-27 cells)	Downregulation of KCNQ1OT1 induces apoptosis of TSCC through intrinsic pathway and regulation of EGFR.	(62)
lncRNA CEBPA-AS1	Up	OSCC	CEBPA	Inhibition of apoptosis	Human (OSCC, n=60), *In vitro* (Tca8113 and Cal-27 cells)	Downregulation of lncRNA CEBPA-AS1 induces apoptosis of OSCC cell through pathway CEBPA/Bcl2 by targeting CEBPA.	(95)
lncRNA H19	Up	OSCC	miR-138	Inhibition of apoptosis	Human (OSCC, n= 42), *In vitro* (HSC-2 and Ca9-22 cells)	lncRNA H19 inhibits apoptosis of OSCC cell through regulating miR-138 expression.	(96)
lncRNA TUC338	Up	TSCC	–	Inhibition of apoptosis	Human (TSCC, n=25) *In vitro* (CAL-27 and SCC-9 cells), *In vivo* (mice)	Downregulation of lncRNA TUC338 induces apoptosis of TSCC cell.	(97)
lncRNA MALAT	Up	TSCC	Wnt/β-catenin signaling pathway	Inhibition of apoptosis	Human (OSCC, n= 42), *In vitro* (HSC-2 and Ca9-22 cells)	MALAT1 suppresses apoptosis of TSCC cells through modulating Wnt/β-catenin signaling pathway.	(98)
LncRNA TUG1		OSCC	Wnt/β-catenin signaling	Inhibition of apoptosis	*In vitro* (Tca8113 and TSCCA cells)	Downregulation of lncRNA TUG1 induces apoptosis of OSCC cells through modulating Wnt/β-catenin signaling pathway.	(99)
lncRNA UCA1	Up	OSCC	miR-184	Inhibition of apoptosis	Human (OSCC, n= 30), *In vitro* (Tca8113-CDDP and TSCCA-CDDP cells)	lncRNA UCA1 knockdown promotes apoptosis rate of OSCC cell by sponging miR-184.	(100)
lncRNA MEG3	Down	OSCC	–	Induction of apoptosis	Human (OSCC, n=83), *In vitro* (Cal27 and SCC15 cells)	lncRNA MEG3 induces apoptosis of OSCC cells	(101)
lncRNA UCA1	Up	OSCC	WNT/β-catenin signaling pathway	Inhibition of apoptosis	*In vitro* (Cal27 and SCC15 cells)	Downregulation of lncRNA UCA1 promotes apoptosis of OSCC cell may be by regulating the WNT/β-catenin signaling pathway.	(102)
lncRNA TTN-AS1	Up	OSCC	miR-199a-3p	Inhibition of apoptosis	Human (OSCC tissue, n=36), *In vitro* (SCC-4 and HSC-3 cells)	Downregulation of lncRNA TTN-AS1 promotes apoptosis of OSCC cells by sponging miR-199a-3p.	(103)
lncRNA TTN-AS1	Up	OSCC	miR-411-3p	Inhibition of apoptosis	Human (OSCC tissue, n=50), *In vitro* (SCC-4 and SCC-9 cells)	lncRNA TTN-AS1 contribute to oral cancer development by inhibition of apoptosis in OSCC cells through regulating miR-411-3p/NFAT5 axis.	(20)
lncRNA TRG-AS1	Up	TSCC	miR-543/YAP1 pathway	Inhibition of apoptosis	Human (TSCC, n= 57), *In vitro* (CAL-27 and SCC-15)	Downregulation of lncRNA TRG-AS1 induces apoptosis of TSCC cells by regulation of miR-543/YAP1 pathway.	(104)
LncRNA LINC00519	Up	TSCC	miR-876-3p	Inhibition of apoptosis	Human (TSCC, n= 52), *In vitro* (SCC-15 and CAL-27)	Downregulation of lncRNA LINC00519 induces apoptosis of TSCC cells by regulation of miR-876-3p/MACC1 axis.	(105)
LncRNA SNHG15	Down	OSCC	miR-188-5p	Inhibition of apoptosis	*In vitro* (SCC-9 and SCC-15)	LncRNA SNHG15 inhibits apoptosis of OSCC cells by sponging miR-188-5p	(19)
LncRNA FER1L4	Up	OSCC	miR-133a-5p	Inhibition of apoptosis	Human (OSCC tissue, n= 45), *In vitro* (SCC-9 and HN4 cells)	Downregulation of LncRNA FER1L4 induces apoptosis of OSCC cells by regulating the miR-133a-5p/Prx1 axis.	(106)

## Circular RNA Biogenesis and CircRNAs-Mediated Programmed Cell Death of Oral Cancer

Circular RNA (circRNA) is a subclass of non-coding RNA (ncRNA) (239–242). After long non-coding RNA (lncRNA) and microRNA (miRNA), it has developed into a new research hotspot in the field of cancer (243). CircRNAs are produced by reverse splicing and are characterized by a closed single-stranded structure and lack of 5′ cap and 3′ polyadenylation (poly(A)) tail, which makes them more stable than lncRNA and miRNA. And circRNAs are highly conserved in eukaryotes, and their expression exhibits tissue- and developmental stage-specific. Numerous studies have revealed that circRNAs have an important effect on the progression and treatment of cancer and play a regulatory role in the tumor microenvironment (TME). Therefore, it suggests that circRNAs may serve as new cancer biomarkers as well as potential therapeutic targets (239, 240, 244, 245).

Back-splicing biogenesis of circRNAs varies from conventional splicing of linear RNAs, according to recent research. They are categorized as intronic-circRNAs, exonic-circRNAs and exon-intron circRNAs (246). Jeck et al. (247). Established two different models considering the circRNAs origination, known as intron-pairing-driven and lariat-driven circularization.Exon circularization is shown to be dependent on flanking intronic complementary sequences and alternate production of inverted repeating Alu pairs, resulting in several circular RNA transcripts being created from a single gene (248). Moreover, RBPs(RNA-binding proteins) function as inhibitors or activators of the circRNAs processing in some circumstances. It has been demonstrated that the generation of up to one-third of common circRNAs are consistently regulated by Quaking(QKI), an alternative splicing factor, and the insertion of QKI motifs are adequate to promote *de novo* synthesis of circRNAs from transcripts normally undergo linear splicing (249). However, by melting the stem’s structure, ADAR1, the double-strand RNA-editing enzyme,inhibits the synthesis of circRNA (250). Nonetheless, the exact mechanism of generation of circRNAs remains poorly clarified.

circRNAs have been identified to be expressed widely in most organisms. The size of circRNAs ranges from <100 to several thousand nucleotides, and are produced by a non-canonical splicing event named back-splicing, during such event the downstream splice-donor site is covalently linked to upstream splice-acceptor site (251). The formed closed-loop structure of circRNAs makes them much more stable than linear RNAs, and their half-life is about 4 times longer than linear RNAs (252). In addition, back-splicing makes circRNAs lack 5’ cap and 3′ tail, which enables them resistant to ribonuclease RNase R. This feature is usually used in experiments to distinguish circRNAs from linear counterparts (253). According to the sequences it contains, circRNA can be divided into three main different types: exon circRNA (EcRNA), intron circRNA (CiRNA) and exon-intron circRNA (EIcRNA) (242, 254). Among them, EcRNA is the most common and is mainly distributed in the cytoplasm, while circRNAs containing introns are distributed in the nucleus. CircRNAs exhibit widespread expression in different species and abundant expression in human tissues, especially in brain. And their sequences are highly conserved, and many studies have detected about 15,000 human circRNAs sequences in mice. Moreover, their expression exhibits in tissue-, cell- and developmental stage-specific manner, and partially dysregulated circRNAs have a close relationship with tumor pathological differentiation, TNM stage and Lei et al, indicating that they may be suitable candidates for cancer biomarkers (253, 255).

In 2000, Hanahan and Weinberg proposed six hallmarks of cancer that result in the progressive conversion of normal cells into cancerous cells (256). Most and perhaps all types of human cancer shared these acquired capabilities, including self-sufficiency in growth signals, evasion of antigrowth signals, resistance to cell death, limitless replicative potential, sustained angiogenesis, tissue invasion and metastasis. In recent years, some circRNAs have been shown to be involved in these properties of cancer (257).

As mentioned above, as a mechanism of cell death, apoptosis is significant for maintaining homeostasis of the internal environment. Tumor cells can often resist apoptosis, so that abnormal cells are not properly cleared. Abundant evidence supports the role of circRNAs in the evasion of apoptosis to promote cancer progression and blunt therapeutic responses. Cancer cells have developed various strategies to limit or evade apoptosis by upregulating anti-apoptotic components (e.g., Bcl-2, Bcl-xL, and Mcl-1) or decreasing the tumor-suppressive function of proapoptotic factors (e.g., Bax). These components involved in apoptosis are broadly inhibited or activated by circRNAs (258). In this section, we summarize the role of circRNAs in apoptosis to determine the relationship between circRNAs and the apoptosis of oral cancer cells (**Table 5**).

**Table 5:** Circular RNAs can regulate cell death pathways in oral cancer.

Circular-RNAs	Expression	Cancer cell	Target of miRNA	Inhibition/Induction of Cell Death	Sample type	Note	Ref
circRNA_100533	Down	OSCC	miR-933	Induction of apoptosis	*In vitro* (CAL‐27 and SCC‐9 cell lines)	circRNA_100533 can induce apoptosis of OSCC cells by sponging miR‐933, and then regulating GNAS expression.	(107)
circBICD2	Up	OSCC	miR-149-5p	Inhibition of apoptosis	Human (Tongue, n= 15; Buccal, n= 5; and gingival, n= 6), *In vitro* (SCC25 and CAL27 cells)	IGF2BP1 inhibits the apoptosis of OSCC cells by regulating the miR-149-5p/IGF2BP1 axis.	(108)
circFNDC3B	Up	OSCC	miR-520d-5p	Inhibition of apoptosis	*In vitro* (CAL27 and SCC15 cells)	Downregulation of circFNDC3B promotes apoptosis of OSCC cells by regulating the miR-520d-5p/SLC7A11 axis	(109)
circ_0109291	Up	OSCC	–	Inhibition of apoptosis	Human (OSCC tissue, n= 51), *In vitro* (CAL27 and SCC4 cells)	Downregulation of circ_0109291 induces apoptosis of OSCC cells.	(110)
circ_0002203	Down	OSCC	–	Induction of apoptosis	Human (OSCC tissue, n=40), *In vitro* (CAL27 and SCC15 cells)	Overexpression of circ_0002203 promotes apoptosis of OSCC cells.	(111)
circ_0055538	Down	OSCC	The p53/Bcl-2/caspase signaling pathway	Induction of apoptosis	Human (OSCC tissue, n= 44), *In vitro* (CAL27 and SCC9 cells)	Overexpression of circ_0055538 induces the apoptosis of OSCC cells by regulating the p53/Bcl-2/caspase signaling pathway.	(112)
circ_0007059	Down	OSCC	–	Induction of apoptosis	Human (OSCC tissue, n=52), *In vitro* (CAL27 and SCC15 cells)	Overexpression of circ_0007059 induces the apoptosis of OSCC cells maybe by remodulation of AKT/mTOR pathway.	(113)
circ_009755	Down	OSCC	–	Induction of apoptosis	Human (OSCC tissue, n=8), *In vitro* (CAL27 and SCC15 cells)	Decrease the expression level of circ_009755 suppresses the apoptosis of OSCC cells.	(114)
circATRNL1	Down	OSCC	miR-23a-3p	Induction of apoptosis	Human (OSCC, tissue, n= 48), *In vitro* (HSC3 and SCC25 cells)	Overexpression of circATRNL1 help to improved sensitivity of OSCC cells to radiotherapy by promotes apoptosis through sponging miR-23a-3p.	(115)
circ_0033144 (CircBCL11B)	Up	OSCC	miR-579/LASP1 axis.	Inhibition of apoptosis	Human (OSCC, tissue, n= 50), *In vitro* (CAL27 and SCC15 cells)	Downregulation of cirCBCL11B promotes the apoptosis of OSCC cells by regulating miR-579/LASP1 axis.	(116)
CircSPATA6	Down	OSCC	miR-182/TRAF6 axis	Induction of apoptosis	Human (OSCC, serum, n= 46), *In vitro* (CAL27 and SCC9 cells)	CircSPATA6 promotes apoptosis of OSCC cell by regulating the miR-182/TRAF6 axis.	(117)
Circ-BICD2	Up	OSCC	miR-296-5p	Inhibition of apoptosis	Human (OSCC tissue, n= 35), *In vitro* (CAL27 and SCC9 cells)	circ-BICD2 can promote apoptosis of OSCC cells might by the regulating the miR-296-5p/TAGLN2 axis.	(118)
Circ-ITCH	Down	OSCC	miR-421	Induction of apoptosis	Human (OSCC tissue, n=57), *In vitro* (SCC6 and HN4 cells)	Overexpression of circ-ITCH promotes apoptosis of OSCC cells through regulating miR-421/PDCD4 axis.	(119)
Circ-PVT1	Up	OSCC	–	Induction of apoptosis	Human (OSCC tissue, n=30), *In vitro* (SCC4 and CAL27 cells)	circ-PVT1 promotes apoptosis of OSCC cells.	(120)
Circ-KIAA0907	Down	OSCC	miR-96-5p	Induction of apoptosis	Human (OSCC tissue, n=59), *In vitro* (HSC6 and OECM1 cells)	circ-KIAA0907 induces apoptosis of OSCC cells through regulating the miR-96-5p/UNC13C axis.	(121)
circKRT1	Up	OSCC	miR‐495‐3p	Inhibition of apoptosis	Human (OSCC tissue, n=50), *In vitro* (Cal‐27 and HSC‐3)	Downregulation of circKRT1 inhibits the apoptosis of CD8+ T cells by regulating by the miR‐495‐3p/PDL1 axis and suggested that suppresses activity of CD8+ T‐cell to induces immune evasion of OSCC cells.	(122)
circ_0000140	Down	OSCC	miR-31	Induction of apoptosis	Human (OSCC tissue, n=56), *In vitro* (Cal-27, HSC-3 and HOK cells)	circ_0000140 promotes apoptosis of OSCC cells by regulation of miR-31/LATS2 axis.	(123)
circRNA_043621	Up	OSCC	–	Inhibition of apoptosis	Human (OSCC tissue, n= 23), *In vitro* (TSCC1 cell)	The circRNA_043621 downregulation leads to induction of apoptosis in OSCC cells.	(124)
circRNA_102459	Down	OSCC	–	Induction of apoptosis	Human (OSCC tissue, n= 23), *In vitro* (TSCC1 cell)	The circRNA_102459 overexpression leads to induction of apoptosis in OSCC cells.	(124)
Circ-HIPK3	Up	OSCC	miR-381-3p	Inhibition of apoptosis	*In vitro* (SCC-4 and SCC-9 cells)	circ-HIPK3 inhibits apoptosis of OSCC cells through regulating miR-381-3p/YAP1 axis.	(125)
CircRNA_10103	Down	OSCC	–	Induction of apoptosis	*In vitro* (OECM1 and HSC3 cells)	Overexpression of circRNA_10103 induces apoptosis of OECM1 and HSC3 cells.	(126)
circ_0001742	Up	TSCC	miR-431-5p	Inhibition of apoptosis	Human (TSCC tissue, n= 30), *In vitro* (CAL27 and SCC4 cells)	circ_0001742 inhibits apoptosis of TSCC cells by regulating the miR-431-5p/ATF3 axis.	(127)
circ_0005379	Down	OSCC	–	Induction of apoptosis	Human (OSCC tissue, n= 37), *In vitro* (SCC25 and CAL27 cells)	Overexpression of circ_0005379 induces apoptosis of OSCC cells might by regulating the EGFR pathway.	(128)
circ_0011946	Up	OSCC	miR-216a	Inhibition of apoptosis	Human (OSCC tissue, n= 30), *In vitro* (SCC25 and CAL27 cells)	Downregulation of expression level of circ_0011946 induces apoptosis of OSCC cells by the regulation of the miR-216a-5p/BCL2L2 axis.	(129)
Circ_0001461	Up	OSCC	miR-145	Inhibition of apoptosis	Human (OSCC tissue, n= 20), *In vitro* (CAL-27 and KB cells)	Circ_0001461 suppresses apoptosis of OSCC cells by regulating miR-145/TLR4/NF-κB axis.	(130)
circCDR1as	Up	OSCC	miR-876-5p	Induction of autophagy and inhibition of apoptosis	Human (OSCC tissue), *In vitro* (OSCC cells)	Upregulation of circCDR1as levels induces autophagy, and inhibits apoptosis in OSCC cells by targeting miR-876-5p.	(131)
circHIPK3	Up	OSCC	miR-637	Inhibition of apoptosis	Human (OSCC tissue, n= 40), *In vivo* (mice) *In vitro* (Tca8113 and SCC-9 cells)	CircHIPK3 inhibits apoptosis of OSCC cells through regulating the miR-637.	(132)
circDHTKD1	Up	OSCC	miR-326	Inhibition of apoptosis	Human (OSCC tissue, n= 56), *In vitro* (SCC9 and Cal27 cells)	circDHTKD1 inhibits apoptosis of OSCC cells by targeting miR-326	(133)
circ_0005623					Human (OSCC tissue, n= 56), *In vitro* (SCC-9 and CAL-27 cells).		
Circ_0002141	Up	OSCC		Inhibition of apoptosis	Human (OSCC tissue, n= 52), *In vitro* (OSCC cells)	Circ_0002141 inhibits apoptosis of OSCC cells by regulating miR-1231/EGFR axis	(134)
circ_0005379	Down	OSCC	miR-17-5p	Induction of apoptosis	Human (OSCC tissue), *In vitro* (SCC15 cells)	Overexpression of circ_0005379 promotes apoptosis of OSCC cells by regulating the miR-17-5p/ACOX1 axis.	(135)
circ‐PKD2	Down	OSCC	miR‐204‐3	Induction of apoptosis	Human (OSCC tissue, n= 56), *In vitro* (SCC15 cells)	circ‐PKD2 induces apoptosis of OSCC cell by suppressing of miR‐204‐3 activity.	(136)
circ_0001971 circ_0001874	–	OSCC	–	Inhibits of apoptosis	*In vitro* (SCC9 cells)	Upregulation of circ_0001971 and circ_0001874 synergistically inhibits apoptosis of OSCC cells.	(137)
Circ_0001971	Up	OSCC	miR-194 and miR-204	Inhibits of apoptosis	Human (OSCC tissue, n= 50), *In vitro* (CAL-27 and SCC9 cells)	Circ_0001971 inhibits apoptosis of OSCC by regulating miR-194 and miR-204.	(138)
Circ_0109291	Up	OSCC	miR-188-3p	Inhibits of apoptosis	Human (OSCC tissue, n= 60), *In vitro* (CAL-27/DDP and UM1/DDP cells)	Circ_0109291 was overexpressed in Cisplatin-resistant OSCC tissues and cells. Downregulation of circ_0109291 promotes apoptosis of OSCC cells by regulating the miR-188-3p/ABCB1 axis.	(139)
circ_0000745	Up	OSCC	miR-488	Inhibition of apoptosis	Human (OSCC tissue, n= 64), *In vitro* (Cal-27 and SCC9 cells)	circ_0000745 inhibits apoptosis of OSCC cells by regulating expression level of miR-488/CCND1.	(140)
Circ-SCMH1	Up	OSCC	miR-338-3p	Inhibition of apoptosis	Human (primary OSCC tissue, n= 62), *In vitro* (SCC-15/DDP and CAL-27/DDP)	Circ-SCMH1 was overexpressed in Cisplatin-resistant OSCC tissues and cells. Circ-SCMH1 contribute to oral cancer development by inhibition of apoptosis in OSCC cells through regulating miR-338-3p/LIN28B.	(141)

The p53 gene is a common tumor suppressor which is involved in cell cycle regulation *via* a variety of pathways and plays an important role in the development of various tumors, including OSCC (259). BAX is a water-soluble protein homologous to BCL-2 and promotes apoptosis. The overexpression of BAX can antagonize the protective effect of BCL-2 and cause cell death. It is located downstream of the p53 signaling pathway and is regulated by the p53 gene (260). Apoptotic protease activating factor-1 (Apaf-1) plays an important role in the mitochondrial apoptotic pathway, and its expression is regulated by the BAX gene. Apaf-1 ultimately mediates caspase family-related proteins, such as caspase-3, which is generally considered the most important terminal cleavage enzyme in apoptosis (261, 262). It has been shown that when hsa_circ_0055538 was overexpressed in OSCC cells (SCC9 and CAL27 cells), the expression levels of p53, p21, BAX, Apaf-1, caspase-3, and cleaved caspase-3 increased, while the expression of Bcl-2 decreased. Thus, upregulation of circ_0055538 promotes cell apoptosis. As well, downregulation of hsa_circ_0055538 in SCC9 and CAL27 cells using siRNA and obtained the opposite results. Hsa_circ_0055538 regulates the malignant biological behavior of oral squamous cell carcinoma through the p53/Bcl-2/caspase signaling pathway (263).

Circular RNA itchy E3 ubiquitin-protein ligase (circ-ITCH) was demonstrated to function as a tumor suppressor in multiple human cancers, such as lung cancer, ovarian carcinoma, and colorectal cancer (264). Recently, Hao et al. (265) investigated the functional role of circ-ITCH in OSCC. They found that the expression level of circ-ITCH was significantly downregulated in OSCC samples and reported the decreased expression of circ-ITCH in OSCC is closely correlated with malignant progression and poor survival in OSCC patients. As well, the results of their study showed that circ-ITCH overexpression suppressed cell proliferation, but promoted apoptosis of OSCC cells *in vitro*. Also, they observed that the increased apoptosis rates of OSCC cells with circ-ITCH overexpression were notably diminished by co-transfection of miR-421, accompanied by the increased expression of Bcl-2 and the decreased levels of Bax, cleaved caspase-3 and PDCD4. Overall, the result of their research showed that circ-ITCH serves as a tumor suppressor in OSCC partly by suppressing cell proliferation and Inducing apoptosis in OSCC by regulating miR-421/PDCD4 Axis (265).

Baculoviral IAP repeat-containing 3 (BIRC3) belongs to the inhibitors of apoptosis (IAP) family of proteins, which suppress proapoptotic signaling. Several studies have reported that BIRC3 may also impair cancer cell susceptibility to chemotherapy (266). Recently, Wang et al. (267) built an apoptotic model with TNF-α, and then they confirmed a circRNA associated with the apoptosis of OSCC cells, circDOCK1 by comparing the expression profile of circRNAs in an apoptotic model with that in untreated OSCC cells. They observed that circDOCK1 is highly expressed in OSCC tissue and cell lines, and circDOCK1 is significantly downregulated in the apoptosis model. In order to further confirm the biological function of circDOCK1 in OSCC, they downregulated circDOCK1 expression using siRNAs and observed that the apoptosis rate increased. With the use of bioinformatics analysis, they surmised that circDOCK1 could serve as a miR-196a-5p sponge. Besides, miR-196a-5p may be implicated in cancer-associated pathways and regulate apoptosis-related genes (268, 269). In order to validate the circDOCK1/miR-196a-5p/mRNA axis, their results revealed that when the expression level of circDOCK1 is downregulated, miR-196-5p was upregulated while BIRC3 was downregulated accordingly. Moreover, when the expression level of miR-196-5p is upregulated, circDOCK1 and BIRC3 were downregulated accordingly, too. At the end of this study, they suggested that circDOCK1 suppresses cell apoptosis *via* inhibition of miR-196a-5p by targeting BIRC3 in OSCC (267).

It has been reported that TLR4 can mediate the immune escape of tumor cells and promote tumor cell to resist drug-induced apoptosis (270). Ai et al. (271) conducted a TNF-a induced apoptosis experiment in OSCC cells. The results of their study showed that TNF-a could induce apoptosis of OSCC cells, which was enhanced by knockdown of circ_0001461. In contrast, overexpression of circ_0001461 lead to inhibit the apoptosis of OSCC cells induced by TNF-a treatment. In addition, they reported that knockdown of circ_0001461 inhibited the expression of TLR4 and NF-kB, while overexpression of circ_0001461 promoted the protein level of TLR4 and NF-kB. Functionally, circ_0001461 directly targeted miR-145 and inhibited the proliferation, migration, and invasion of OSCC cells. TLR4, a direct target of miR-145, served as the key mediator of circ_0001461 function by promoting the resistance of immune induced apoptosis of OSCC cells. Together, their results suggested that circ_0001461 might promote OSCC cells to resist TNF-a induced apoptosis through TLR4 mediated NF-kB pathway (271).

Cetuximab is a recombinant human murine chimeric IgG1 monoclonal antibody, which has high affinity for EGFR, inhibits cell cycle progression, and induces tumor cell apoptosis by specifically binding to the extracellular EGFR domain. This reduces the production of MMPs and vascular endothelial growth factors, and inhibits tumor invasion and metastasis. Cetuximab has shown good clinical efficacy and tolerability for EGFR expression in head and neck cancers (272, 273). However, unresolved issues with this drug persist, such as acquired treatment resistance caused by mutations in KRAS and BRAF, both of which participate in the EGFR signaling pathway (274). It has been reported that ncRNAs can be regulated cetuximab sensitivity in cancer cells (275–277). Since cetuximab is a commonly used anticancer drug for OSCC treatment, recently Su et al. (278) performed a drug treatment experiment to investigate the effect of hsa_circ_0005379 on OSCC cell viability. In their study, apoptosis rates in hsa_circ_0005379 overexpression cells were measured by annexin V-FITC/PI dual-label flow cytometry and flow cytometry to detect apoptosis in different treatment groups of SCC25 and CAL27 (OSCC cell lines) was used. They found that early apoptotic rates in the mock group were 0.31 and 0.43% in SCC25 and CAL27 cells, respectively, while early apoptotic rates in the hsa_circ_0005379 group were 1.12 and 0.91% in SCC25 and CAL27 cells, respectively. Early apoptotic rates in the mock + cetuximab group were 17.88 and 15.22% in SCC25 and CAL27 cells, respectively, while early cell apoptotic rates in the circ_0005379 + cetuximab group increased to 38.35 and 35.77% in SCC25 and CAL27 cells, respectively. Their experimental results showed that changes in early apoptotic rates are not obvious in cells after high hsa_circ_0005379 expression in SCC25 and CAL27 cell lines. However, when cetuximab was added after overexpressing hsa_circ_0005379 in SCC25 and CAL27 cells, the early apoptotic rate of the cells significantly increased to 38.35 and 35.77%, respectively. This study indicated that high hsa_circ_0005379 expression increases cetuximab sensitivity and provides a new potential target for OSCC anticancer drugs design in the future (278). The occurrence of cisplatin (DDP) resistance in oral squamous cell carcinoma (OSCC) is a major challenge for OSCC treatment. Gao et al. reported that Circ_0109291 was higher expressed in DDP-resistant OSCC tissues and cells, and its knockdown suppressed resistance and enhanced the apoptosis of OSCC cells. They suggested that circ_0109291 could promote the DDP resistance and inhibit apoptosis of OSCC, and silenced circ_0109291 might be a key step to inhibit OSCC resistance (279). In another study, Tan and colleagues (280) found that circ_0001971 knockdown promoted chemosensitivity (DDP) and apoptosis in OSCC cells by interacting with miR-194 and miR-204 (280). Such studies show the circRNAs can serve as critical player in OSCC cell resistance and the functions of the circRNAs provide insight into their roles in oral cancer chemotherapy resistance. More studies about circRNAs-mediated PDC of oral cancer are listed in **Table 5**.

**Figure 3:**
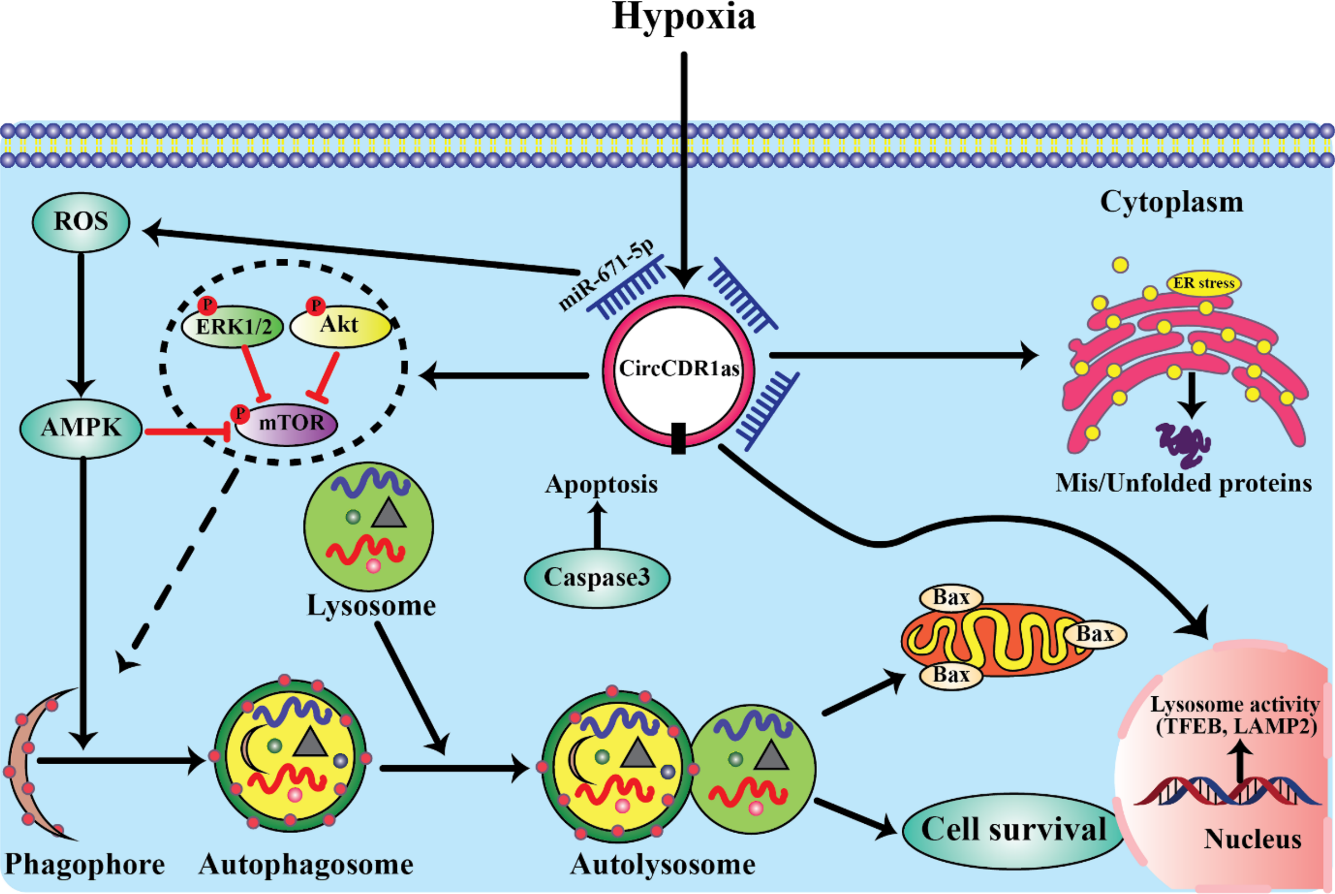
The mechanism underlying the regulation of autophagy.

Recent studies have shown that autophagy plays a critical role in the occurrence of tumors and malignant transformation (101, 165, 281). In advanced stage tumors, cancer cells survive under low-nutrition and hypoxic conditions by inducing autophagy due to cancer cells have higher bioenergy requirements and nutritional needs than normal cells (165). The elucidation of the association between autophagy and poor survival in various cancers, suggested that autophagy may serve as a marker for both diagnostic and clinicopathological characteristics (282, 283). Thus, understanding the signaling pathways involved in the regulation of autophagy as well as its biological functions in OSCC represents new directions in the development of anticancer therapeutic strategies. Recently, Gao et al. (284) validated the functional roles of circCDR1as in regulation of autophagy in OSCC cells and further investigated how circCDR1as contributed to cell survival *via* up-regulating autophagy under a hypoxic microenvironment by using combination of human tissue model, *in vitro* cell experiments and *in vivo* mice model. They found that overexpression circCDR1as not only promoted OSCC cells proliferation *in vitro* and the growth of implanted tumors *in vivo* but also stimulated cells autophagy. The effects of circCDR1as on cellular autophagy contributed to OSCC progression and development. As well, hypoxia promoted the expression level of circCDR1as in OSCC cells and elevated autophagy. In addition, circCDR1as further increased hypoxia-mediated autophagy by targeting multiple key regulators of autophagy. They revealed that circCDR1as enhanced autophagy in OSCC cells *via* inhibition of rapamycin (mTOR) activity and upregulation of AKT and ERK½ pathways. Overexpression of circCDR1as enhances OSCC cells viability, endoplasmic reticulum (ER) stress, and inhibited cell apoptosis under a hypoxic microenvironment. Moreover, circCDR1as is promoted autophagy in OSCC cells by sponging miR-671-5p. Collectively, the results of their research revealed that high expression of circCDR1as enhanced the viability of OSCC cells under a hypoxic microenvironment by promoting autophagy, suggesting a novel treatment strategy involving circCDR1as and the inhibition of autophagy in OSCC cells (284) (**Figure 3**). Consistent with this studies, Cui et al. (285) reported overexpression of circCDR1as promoted autophagy, cell cycle, proliferation, and metastasis and repressed apoptosis in OSCC cells. CircCDR1as directly targeted miR-876-5p and miR-876-5p interacted with SLC7A11. MiR-876-5p overexpression was reversed the effects of circCDR1as elevation on OSCC cell autophagy, cell cycle, growth, motility, and apoptosis. In addition, circCDR1as knockdown blocked tumor growth *in vivo*. They suggested that circCDR1as acted as an oncogene in OSCC progression through elevating SLC7A11 by targeting miR-876-5p (285).

## Conclusion

Apoptosis and autophagy are physiologically necessary pathways that are vital for cell homeostasis and as one of the major dysregulated processes in the carcinogenesis of oral cancer has been shown to be regulated by ncRNAs. In the current review, we have explained the impact of miRNAs, lncRNAs and circRNAs on apoptosis and autophagy in oral cancer. These ncRNAs interact with PI3K/Akt, NF-κB, Wnt/β-catenin, EGFR, TGF-β and other cancer-related pathways (**Tables 3**–**5**). Therefore, they not only regulate cell death pathway, but also influence other aspects of oral carcinogenesis.

Manipulation of expression of apoptosis-regulating circRNA, lncRNAs and miRNAs represent a strategy for combating carcinogenesis as well as resistance to chemo/radiotherapy. Some of the apoptosis-regulating circRNA/miRNA and miRNAs/lncRNAs have been shown to influence prognosis of lung cancer. The observed correlation between their expression and patients’ survival is due to their impact on disease progression as well as response of patients to EGFR inhibitors and chemotherapeutic agents.

An acknowledged route of function of lncRNAs and circRNA in the regulation of apoptosis in oral cancer is their impact on expression of miRNAs. In fact, they can sponge or sequester miRNAs and release miRNA targets from their inhibitory effects. Circ-SCMH1/miR-338-3p, circ_0109291/miR-188-3p, circ_0001971/miR-194/miR-204, circ_0001874/miR-296, circ‐PKD2/miR‐204‐3p, circ_0001461/miR-145, circ_0011946/miR-216a-5p, lncRNA-KCNQ1OT1/miR‐185‐5p, lncRNA RP5-916L7. 2/miR-328, LncRNA MEG3/miR‐548d‐3p, and LINC00958/miR-4306 are examples of circRNAs/miRNAs and lncRNAs/miRNAs interactions with verified roles in the control of oral cancer cells apoptosis. Based on the importance of apoptotic pathways in determination of response of oral cancer patients to conventional as well as targeted therapies, identification of the impacts of ncRNAs on apoptosis and prior profiling of these ncRNAs in clinical samples would help in prediction of response of patients to each therapeutic regimen and design of personalized treatment strategies. The advent of high throughput sequencing strategies has facilitated conduction of this approach in the clinical settings. Finally, the possibility of ncRNAs tracing in the peripheral blood of patients has opened a new opportunity for early detection of emergence of resistance to conventional or targeted therapies and modulation of therapeutic regimens to enhance the survival of affected individuals.

## Author Contributions

MT involved to conception, design, statistical analysis and drafting of the manuscript. LE and , AAS contributed to data collection and manuscript drafting. All authors contributed to the article and approved the submitted version

## Conflict of Interest

The authors declare that the research was conducted in the absence of any commercial or financial relationships that could be construed as a potential conflict of interest.

## Publisher’s Note

All claims expressed in this article are solely those of the authors and do not necessarily represent those of their affiliated organizations, or those of the publisher, the editors and the reviewers. Any product that may be evaluated in this article, or claim that may be made by its manufacturer, is not guaranteed or endorsed by the publisher.
